# BrainInsights: a comprehensive framework for pre-processing, analysis, and interpretation of neuroimaging data using traditional statistics and machine learning

**DOI:** 10.3389/fninf.2026.1760583

**Published:** 2026-04-15

**Authors:** Mageshwar Selvakumar, Andrea Mendez Torrijos, Laura Cristina Konerth, Stefanie Horndasch, Raja Atreya, Arnd Doerfler, Georg Schett, Juergen Rech, Andreas Hess

**Affiliations:** 1 Institute of Pharmacology and Toxicology, Friedrich-Alexander-Universität Erlangen–Nürnberg, Erlangen, Germany; 2 Department of Child and Adolescent Psychiatry and Psychotherapy, Medical School and University Medical Center OWL, Protestant Hospital of the Bethel Foundation, Bielefeld University, Bielefeld, Germany; 3 Department of Internal Medicine 1, Friedrich-Alexander-Universität Erlangen–Nürnberg and Universitätsklinikum Erlangen, Erlangen, Germany; 4 Department of Neuroradiology, Friedrich-Alexander-Universität Erlangen–Nürnberg and Universitätsklinikum Erlangen, Erlangen, Germany; 5 Department of Internal Medicine 3—Rheumatology and Immunology, Friedrich-Alexander-Universität Erlangen–Nürnberg and Universitätsklinikum Erlangen, Erlangen, Germany; 6 FAU NeW—Research Center for New Bioactive Compounds, Friedrich-Alexander- Universität Erlangen–Nürnberg, Erlangen, Germany

**Keywords:** clinical translation, interpretable models, machine learning framework, multi-modal neuroimaging, reproducibility, statistical analysis

## Abstract

Neuroimaging presents us with an in-depth understanding about brain structure and function, yet the data complexity poses significant analytical challenges. Current frameworks suffer from issues such as scalability, poor integration with traditional statistics and a need for a programing background, which hinder researchers from focusing on neuroscience questions. To address these limitations, we present BrainInsights, an integrated and automated GUI-based pipeline ecosystem designed to facilitate the analysis of multi-modal or multi-parametric neuroimaging data in a flexible way. The framework comprises three core tools: MARIA (MAgnetic Resonance Imaging data Analysis and inspection tool) for data inspection and hypotheses testing, ML Pipeline for automated feature selection and model construction, and ML DaViz for model evaluation and bio-signature generation. Deployed as a singularity container, the system ensures reproducibility and scalability across computing environments. We validated BrainInsights using diverse datasets, including multi-parametric MRI studies of Anorexia Nervosa, Crohn’s disease, and Rheumatoid Arthritis. Specifically, the framework distinguished young Anorexia Nervosa patients from controls with a balanced accuracy of 65%, while in the PreCePRA trial, it predicted Rheumatoid Arthritis treatment response with a balanced accuracy of up to 95.4% using functional pain markers. The results demonstrate the ability of the framework to achieve high separation of subgroups and treatment success and additionally bridge hypotheses-driven statistical analysis with data-driven machine learning analysis. By enabling interpretability tools like SHAP, BrainInsights empowers researchers to move beyond “black-box” modeling to uncover stable, biologically plausible bio-signatures. Ultimately, this framework aids in accelerating the translation of complex neuroimaging data into meaningful clinical insights.

## 1 Introduction

Neuroimaging is an essential armamentarium in neuroscience, helping researchers understand the brain’s complexity and its intricate network of neurons. The different neuroimaging modalities like magnetic resonance imaging (MRI), positron emission tomography (PET), and electroencephalography (EEG) provide us unique and distinct perspectives on the brain’s anatomical structure, function, and connectivity. These tools are powerful for neuroscience research and are instrumental in studying both brain function and a range of neurological and psychiatric conditions (Biessmann et al., 2011; Tulay et al., 2019). Multi-modal imaging, i.e., integrating different neuroimaging modalities, can provide a more comprehensive understanding of the brain and enhance the insights gained from neuroimaging studies (Biessmann et al., 2011). However, multi-modal neuroimaging data analysis comes with a significant challenge: the resulting dataset is more complex and thus requires a more sophisticated analysis to process and extract meaningful information.

Current machine learning (ML) frameworks used in neuroimaging such as WEKA (Frank, 2005), RapidMiner (Mierswa et al., 2006), among others, face several limitations. These platforms often struggle with scalability when dealing with large neuroimaging datasets and suffer from reproducibility issues when analyses are moved between computing environments. Furthermore, they typically require significant programing expertise, posing a barrier for researchers who wish to focus on neuroscience questions rather than technical implementation. A prominent deficiency is the poor integration of traditional statistical tests with modern ML methods, and a notable hurdle is the absence of integrated and intuitive visualization tools—like chord or connectome plots—to convey findings effectively.

This paper presents BrainInsights, an integrated GUI-based framework designed to overcome the above challenges and facilitate a flexible and systematic analysis of multi-parametric/multi-modal neuroimaging data. BrainInsights is a collection of three powerful tools:

- MARIA (a preliminary data analysis and visualization tool).
- ML Pipeline (machine learning framework comprising various algorithms and feature selection algorithms that run on multi-modal/multi-parametric or subsets of data).
- ML DaViz (an evaluation tool for different feature selection algorithms and classification algorithms obtained from the machine learning framework).

It is important to clarify that BrainInsights is designed to operate on derived, feature-extracted neuroimaging data from tabular formats rather than raw voxel-level images. The framework does not perform initial raw image pre-processing such as motion correction, skull stripping or registration—but instead ingests the statistical outputs from established pipelines like Freesurfer, VBM or DBM.

Together these tools seamlessly address the issues of traditional statistical analysis, hypothesis generation and testing, feature selection, building machine learning models, and visualization of individual features and classification results. In addition to providing advanced data exploration and hypothesis testing, this novel framework enables the generation of interpretable and actionable insights from neuroimaging data. It bridges the gap between traditional statistical analysis and modern machine learning (ML) techniques in an easy-to-use GUI-based interface.

The primary goal of BrainInsights is to simplify the data analysis process and enable researchers to uncover hidden patterns in data and generate characteristic “bio-signatures” for specific cohorts and/or treatment success. From this perspective, a bio-signature is a distinct and measurable set of features—derived from brain structure, function or clinical data—that collectively serves as a signature for a specific condition, such as differentiating treatment responders from non-responders. Depending on the research question, it allows for traditional hypothesis-driven statistical assessments but also provides methods of data-driven evaluations, thereby leading to new hypotheses and findings. The framework has been designed to be adaptable to incorporate multiple neuroimaging modalities and parameters as well as clinical data for multi-center, multi-measurement (repeated measurements at different time points) studies for animal but also human data. The framework provides the ability to test the datasets with multiple feature selection and ML classification algorithms, cross-evaluate them, and establish bio-signatures, e.g., for different diseases or to differentiate responders from non-responders to drugs. Inspection and interactive evaluation of individual features, atlases, or modalities are possible through various plots available or as tables. MARIA and ML DaViz are GUI-based while the ML Pipeline is completely automated via scripts. High-quality output is possible as images and structured text. The framework is implemented and deployed as a singularity container, making it reproducible, platform-independent, and easily scalable on HPC clusters.

To demonstrate the capabilities of BrainInsights, we conducted extensive experiments using several datasets, including a multi-centric multi-parametric MRI PreCePRA dataset with additional clinical data (Hess et al., 2025; Schenker et al., 2021), a multi-parametric MRI Anorexia dataset (Mendez-Torrijos et al., 2024), a multi-parametric MRI Crohn’s disease study (Hess et al., 2015), among others. The results exemplify our framework’s versatility and robustness and highlight its potential to advance the neuroimaging data analysis landscape for both human and animal studies.

## 2 Framework design, data structure, and analysis workflow

### 2.1 BrainInsights overview

The BrainInsights workflow begins by ingesting tabular data from common formats such as Excel, CSV, or SYLK files from any standard data analysis pipeline of the specific data in use. The framework automatically converts these inputs into a unified, structured format suitable for use across all its components.

While designed for neuroimaging, the architecture is data-agnostic and can process any tabular data, including detailed clinical or demographic information. A key and quite unique feature enhancing this flexibility is the use of an optional external group assignment file. This allows users to dynamically define or update subgroups for analysis—for example, based on age, gender, handedness, treatment success, or combinations thereof. New group definitions can be added as columns, enabling efficient re-analysis of the data without needing to re-import or re-process the entire dataset.

**FIGURE 1:**
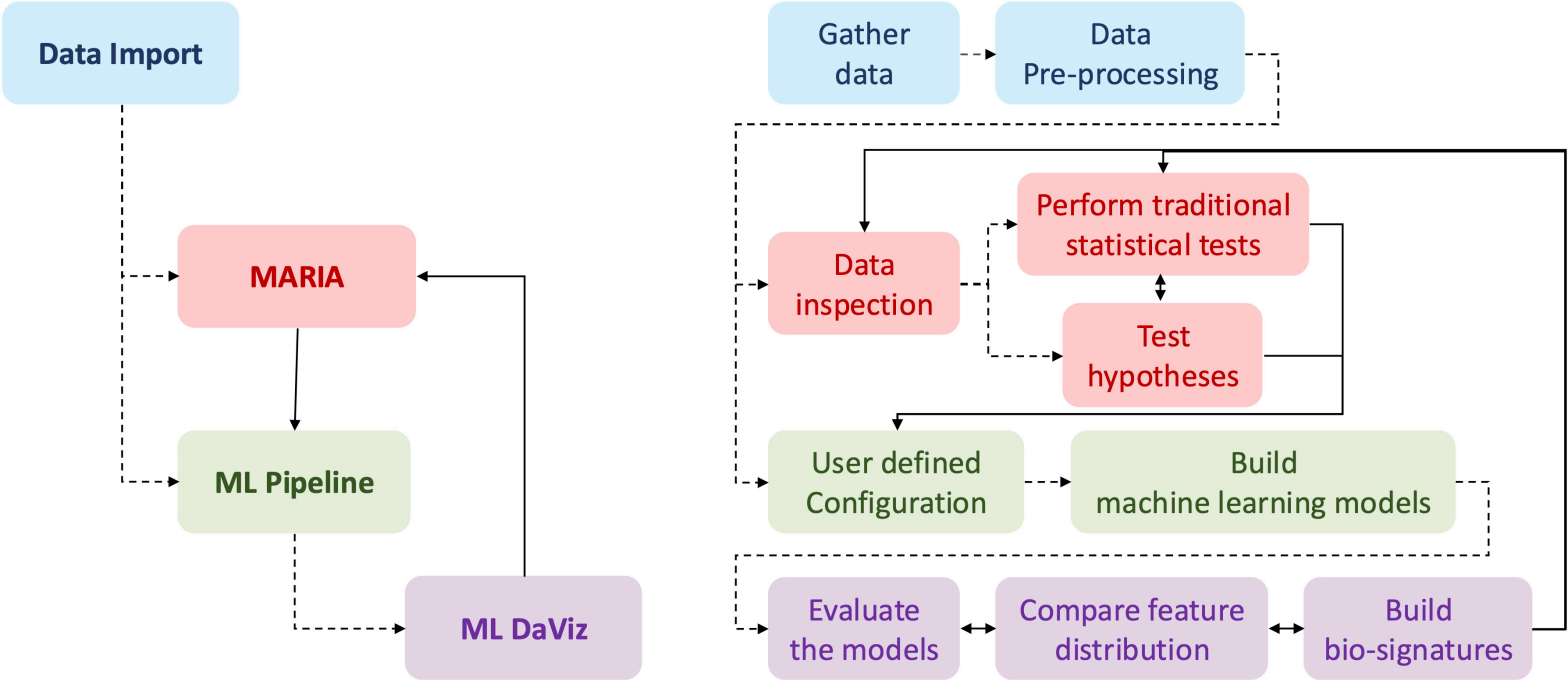
Workflow of brain insights framework: the diagram illustrates the integrated structure of the framework. The left side outlines the major components to provide higher-level overview, while the right side details the key processes within each module. Dotted lines indicate the flow of data, and the solid lines represent the conceptual workflow. The framework starts with data import and pre-processing (blue), and branches into three main modules: MARIA for statistical analysis and hypothesis testing (red), ML Pipeline for machine learning model construction (green), and ML DaViz for model evaluation and bio-signature generation (purple).

**FIGURE 2:**
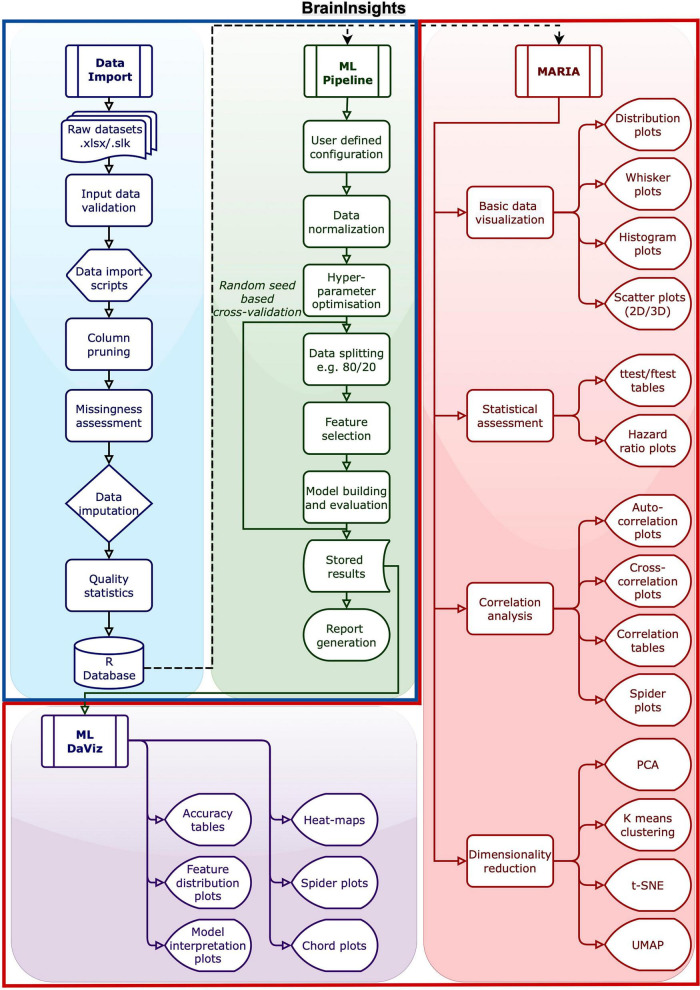
BrainInsights overview: a detailed flowchart of each of the four parts of the framework. The Data Import section imports raw datasets, applies necessary imputation and filtering and saves the data. MARIA section can use this data for inspection and testing hypotheses in the form of plots and tables. The ML Pipeline uses the data from the data import step to build ML models after feature selection. The results from ML Pipeline can be inspected and analyzed with ML DaViz. The pipelines inside the larger blue box (Data Import and ML Pipeline) are accessible via scripts and may take longer to run, fitting a “do it and go home” batch process. The tools in the large red box (MARIA and ML DaViz) are interactive and is part of a GUI, allowing for easy exploration and testing.

Figure 1 provides a schematic overview of the BrainInsights framework. The specific processes within each of the four color-coded components are detailed in the flowchart in Figure 2.

- DATA IMPORT (Blue Section): Handles the initial data import and pre-processing steps, including data cleaning and harmonization.
- MARIA (Red Section): MAgnetic Resonance Imaging data Analysis and inspection tool is used for data inspection and hypothesis testing. It performs traditional statistical analyses, group comparisons, dimensionality reduction, and clustering on pre-processed data.
- ML PIPELINE (Green Section): This is the core Machine Learning engine. It automates key steps including data pre-processing, filtering, hyperparameter tuning, feature engineering, and building predictive models.
- ML DaViz (Purple Section): Machine Learning Analysis and Data Visualization tool takes the output from the ML Pipeline to evaluate model performance and interpret results. The workflow allows users to compare feature distributions and build bio-signatures using a variety of visualizations including violin, chord and spider plots to highlight the most significant features in the data.

The specific input and output specifications for each of these modular components are detailed in Supplements Table 1.

### 2.2 Target user profiles and accessibilities

BrainInsights is designed to be accessible to a wide range of researchers, regardless of their programing background. To achieve this, we focus on two distinct ways people interact with the framework.

#### 2.2.1 For clinical and neuroscience researchers

MARIA and ML DaViz modules are designed to focus primarily on science rather than on writing code. These components are built as interactive Shiny-based GUIs that function entirely through a “point-and-click” interface. A detailed visual guide of the GUI navigation and functional modules is provided in the supplement methods (cf. Supplementary Figure 1). This allows researchers to perform traditional statistics, visualize data distributions, create hypotheses, e.g., by dimensionality reduction, and interpret machine learning results without needing any programing expertise.

#### 2.2.2 For data scientists and ML practitioners

For users who need to scale up their analysis, the ML Pipeline provides an automated approach for batch processing. While the specific analysis steps are managed through a single Excel configuration file, executing the pipeline does require a basic comfort level with a Linux terminal. This setup is designed to run with singularity containers, making it easy to manage large-scale jobs on High-Performance Computing (HPC) clusters.

### 2.3 BrainInsights environment and workflow

All tools and scripts were developed using the R programing language V4.3.1 (R Core Team, 2021). The utilized R packages are listed in Table 1. To ensure that the results are reproducible and the software remains platform-independent, the entire implementation is provided as a Singularity container (Kurtzer et al., 2017). This allows the tools to scale seamlessly from local workstations to High-Performance Computing (HPC) clusters.

**TABLE 1:** R Packages.

Data manipulation	Dplyr (Wickham et al., 2018), tidyr (Wickham et al., 2024), stringr (Wickham, 2023)
Visualization	ggplot2 (Wickham, 2016), plotly (Sievert, 2020), heatmaply (Galili et al., 2017), fmsb (Nakazawa, 2024), ggbeeswarm (Clarke and Sherrill-Mix, 2024), circlize (Gu et al., 2014)
Imputations	VIM (Kowarik and Templ, 2016), mice (Zhang, 2016)
Dimensionality reduction, machine learning and feature selection	Caret (Kuhn, 2008), Boruta (Kursa and Rudnicki, 2010), mixOmics (Rohart et al., 2017), Recursive Feature Elimination (Chen and Jeong, 2007), RandomForest (Liaw and Wiener, 2002), Hmisc (Harrell, 2024), Rtsne (Krijthe et al., 2018), uwot (Melville, 2025), e1071 (Dimitriadou, 2009), rSimca (Filzmoser and Todorov, 2013; Filzmoser and Vlaskova, 2022), rferns (Kursa, 2014), XGBoost (Chen et al., 2019), catboost (Dorogush et al., 2018), nnet (Ripley et al., 2016)
Parallel processing	Purrr (Wickham and Henry, 2023), foreach (Microsoft and Weston, 2022), doParallel (Corporation and Weston, 2022)
Miscellaneous	Shiny (Chang et al., 2023)

Overview of primary R libraries utilized within the BrainInsights ecosystem for data processing, machine learning and visualization.

The workflow is designed to move from script-based data preparation to interactive analysis:

- Data Ingestion: While the analysis modules are interactive, the initial data import and pre-processing steps are handled via R scripts. This means a basic understanding of programing is required at the outset to convert raw, feature-extracted neuroimaging data into the framework’s structured format. Additionally, as BrainInsights is dedicated to downstream analysis, it is expected that raw image processing—including motion correction, skull stripping and registration—has been performed externally using modality-specific pipelines such as fMRIPrep, Freesurfer or VBM. Once this is complete, the data works seamlessly across MARIA and ML Pipeline modules.
- The Machine Learning Cycle: Machine learning within the framework is a two-stage process. First, the ML Pipeline executes the computationally heavy tasks—such as feature engineering, hyperparameter tuning, and model building. Once processed, results are explored through ML DaViz and for visual interpretation of bio-signatures.

### 2.4 Software verification and quality assurance

To ensure the reliability of the framework’s core analytical engine, we implemented a structured verification process. Individual R functions—specifically those responsible for data cleaning, curation, and missing value imputation were verified using the testthat (Wickham, 2011) package. The unit testing suite was designed to confirm that the software handles edge cases, such as unexpected non-numeric entries or features with high missingness, without compromising the integrity of the analysis pipeline. Code coverage was done using covr (Hester, 2023) package and the analysis demonstrates a high degree of testing density across the framework. The Data Import module achieves 95.11% coverage, MARIA 93.08%, ML DaViz 93.32%, and the ML Pipeline 90.11%.

Furthermore, the deployment of BrainInsights within a singularity container functions as environment-level verification. By freezing the versions of all R package dependencies, we eliminate dependency drift and ensure that the software behaves identically across different computing platforms, from local machines to HPC clusters. This is complemented by the inclusion of a sample configuration file (provided as a converted PDF of the native YAML settings), which details the exact hyperparameters, random seeds, and algorithms used, allowing researchers to replicate the reported classification outcomes precisely. This is further supported by the iterative validation described in Case Study 3, where ML Pipeline outputs are visually cross-checked in MARIA to identify potential technical artifacts or systematic processing errors.

### 2.5 Data import

#### 2.5.1 Data structure and compatibility

BrainInsights is designed to be data-agnostic, ingesting processed tabular data instead of raw voxel-level images or region by time-series matrices. The framework assumes that raw neuroimaging scans have already been processed using modality-specific software (e.g., Freesurfer, VBM or fMRIPrep) to generate derived features. To accommodate the wide range of supported multi-modal and multi-parametric imaging and the numerous atlases available for each modality, as in the Freesurfer (Fischl and Dale, 2000; Fischl et al., 2002) for MRI data—we utilize three primary organizational templates for input:

- Region-based Metric Tables: This format handles anatomical measurements (VBM, Ashburner and Friston, 2000); DBM, Davatzikos et al., 1996, Freesurfer), DTI, and functional summary maps like ALFF and ReHo. Each row corresponds to a regional label from a specific atlas, while columns represent extracted measures such as volume, surface area, or mean intensity. For resting-state fMRI, the framework expects these pre-calculated regional summary statistics or flattened connectivity vectors rather than raw nodal time-series.
- Graph Theoretical Parameters: Network-based data is organized into two distinct structures. Global metrics are formatted with rows representing a threshold or density range and columns representing network-wide properties like small-worldness or total edge count. Nodal parameters follow the region-based format, where rows are brain structures and columns are local metrics like clustering coefficients or betweenness centrality.
- Clinical and Demographic Data: These Excel files (.xlsx) can be imported with features as rows (one file per participant) or as combined tables where columns represent specific variables like age, blood parameters or other clinical scores.

Regardless of the initial input template, the framework automatically flattens and concatenates these files into a unified subject-by-feature matrix. The specific input and output requirements for utilizing each module as a standalone tool are summarized in Supplementary Table 1. The framework utilizes an early fusion strategy, where cross-modal features are integrated into a single high-dimensional space prior to feature selection and model training. This design choice enables the algorithms to identify complex, non-linear interactions across different imaging modalities and clinical markers that might be overlooked if each subset was analyzed in isolation. While effective for identifying unified bio-signatures, we are evaluating the implementation of late fusion or hybrid strategies for future iterations to effectively handle datasets characterized by asymmetries in feature dimensionality or varying information density across modalities. In this structured output, each row represents a single participant and each column represents a unique “feature” defined by the combination of the Modality/Pipeline, atlas, brain structure and measure.

For example:

- Freesurfer + aseg atlas + Hippocampus + Volume
- DTI + GM atlas + any structure + Intensity

This standardized schema ensures that the components of BrainInsights remain modular; while they are part of an integrated ecosystem, each tool can be used independently. The structured data can be saved with the lightweight RDS (R data structure) or high-speed Feather formats.

#### 2.5.2 Data pre-processing pipeline

The data pre-processing pipeline begins with a quality assessment followed by a data curation step designed to correct errors, handle NAs and NaN values, and remove unexpected non-numeric entries, thereby ensuring data integrity. Missing data is managed by a two-step process. Specific recommendations for handling various rates of missingness are detailed in Supplementary Table 2. First, as an optional step, to prevent introducing analytical bias, features with a high percentage of missing values (e.g., > 30%) can be dropped. Second, for the remaining features, several imputation methodologies are available. While the choice is user-dependent, the pipeline provides guidance, generally recommending simpler methods such as mean/median imputation for lower rates of missingness and more advanced methods such as K-Nearest Neighbors imputation based on feature similarity and Multivariate Imputation by Chained Equations (MICE) for more complex scenarios.

Following imputation, optional data scaling and normalization options are available. Methods such as standard scaling, robust scaling, min-max scaling, logarithmic or box-cox transformations are available. The mathematical formulas and typical applications are summarized in Supplementary Table 3. Additionally, a specialized within-subject normalization option is available that can be particularly helpful for longitudinal data analysis. This approach applies the selected scaling method to the repeated measurements within each subject, controlling for individual baseline differences. In cases where a subject has only a single measurement, normalization is instead performed using the group average. Furthermore, all imputations and scaling methods can be applied flexibly, either across the entire dataset or within various subgroups.

#### 2.5.3 Practical implementation guidelines

BrainInsights was designed keeping practical considerations in mind to streamline research workflows and robust analysis as outlined below.

##### 2.5.3.1 Standard workflow procedures

The data import process is straightforward. The data import pipeline converts raw feature-extracted neuroimaging data from tabular data different formats into a standardized R data structure. This standardized format works seamlessly on other tools within the BrainInsights framework. Additionally, an external group assignment file available in excel format aids in dynamic group analyses without reloading data repeatedly.

##### 2.5.3.2 Troubleshooting protocols

The data import pipeline is equipped with an extensive logging feature that helps troubleshooting issues with data. During the import process, detailed quality statistics are generated to help flag potential issues early. After import, users can quickly inspect the data using MARIA to visually detect outliers or unexpected distributions, issues that may point to data quality problems, as demonstrated in Case Study 3.

##### 2.5.3.3 Best practical recommendations

For optimal performance, we recommend the following:

- Ensure consistency across all raw datasets in terms of structure and feature-extracted methods. When and where possible, register data to a similar space before the feature extraction process, as it can be ineffective when each parametric neuroimaging data is in a different space.
- A user-defined but consistent naming convention has to be guaranteed across all data sources.
- Choose imputation methods carefully to avoid introducing bias in the datasets. For low missing rates, simple imputation methods may suffice. However, as the missing rates increase advanced methods like MICE can provide more accurate estimates.
- Leverage the group assignment file for efficient and flexible subgroup analyses without having to reload and reprocess datasets.

### 2.6 MARIA (MAgnetic Resonance Imaging data Analysis and inspection tool)

MARIA is an R/Shiny-based GUI tool designed for the comprehensive analysis and visualization of feature-extracted neuroimaging data. It integrates data inspection, traditional statistical analysis, and advanced visualization techniques into a single, user-friendly platform.

#### 2.6.1 Data Inspection and visualization

##### 2.6.1.1 Basic visualization plots

To facilitate understanding of the dataset’s structure, MARIA includes standard plot types such as line plots, histograms, density plots, scatter plots (2D/3D), bee swarm plots and whisker plots. These visualizations are widely used in neuroimaging research for examining temporal changes, distributions, relationships between variables, and between-group differences.

These tools allow users to quickly identify patterns, outliers, or potential issues in the data.

**FIGURE 3:**
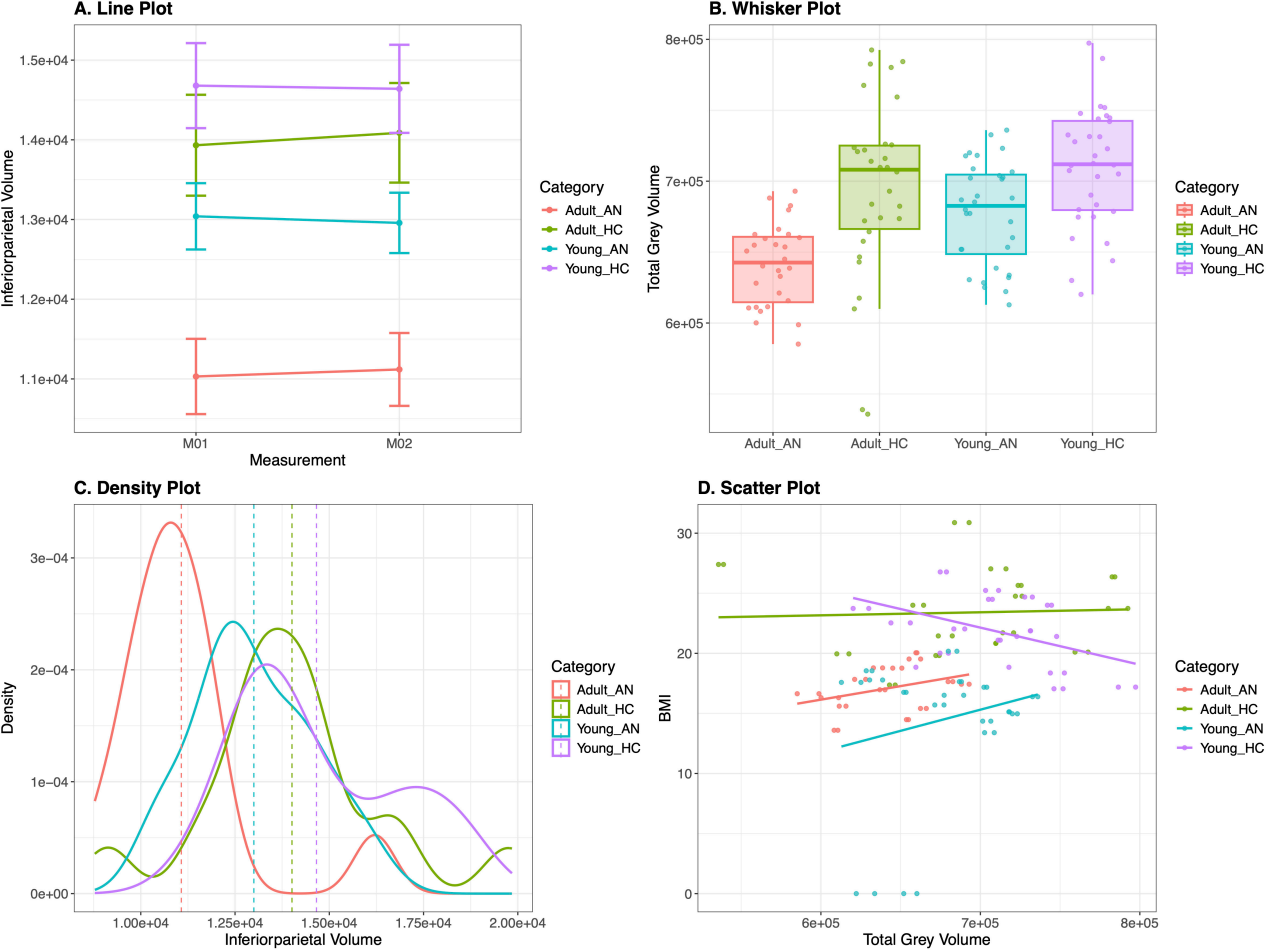
Basic visualization plots in MARIA. In **(A)** a line plot displaying mean values with standard error of measurement (SEM) of inferior parietal volume across two measurement points (M01 and M02) for all four groups. **(B)** The density distribution of inferoparietal volume measurements across all groups, highlighting the different patterns between adult and young subjects with and without anorexia. **(C)** Whisker plots comparing total gray volume distribution across the four subject categories (Adult_AN, Adult_HC, Young_AN, Young_HC), with individual data points overlaid to show the spread within each group. **(D)** displays a scatter plot with total gray volume on the x-axis and BMI on the y-axis, with regression lines for each group showing the relationship between brain volume and BMI.

Figure 3 showcases examples of these plots.

##### 2.6.1.2 Advanced plots

Beyond basic inspection, MARIA enables more integrated views assessing the relationships across multiple features or dimensions:

- Correlation Plots (Auto and Cross-Correlation): Used to examine intra- and inter-dataset relationships (e.g., structural–structural or structure–clinical parameter associations).
- Spider (Radar) Plots: Interactive visualizations for comparing multiple metrics (e.g., mean, percentage change) across selected features or ROIs, useful in summarizing outputs from statistical or machine learning pipelines.

**FIGURE 4:**
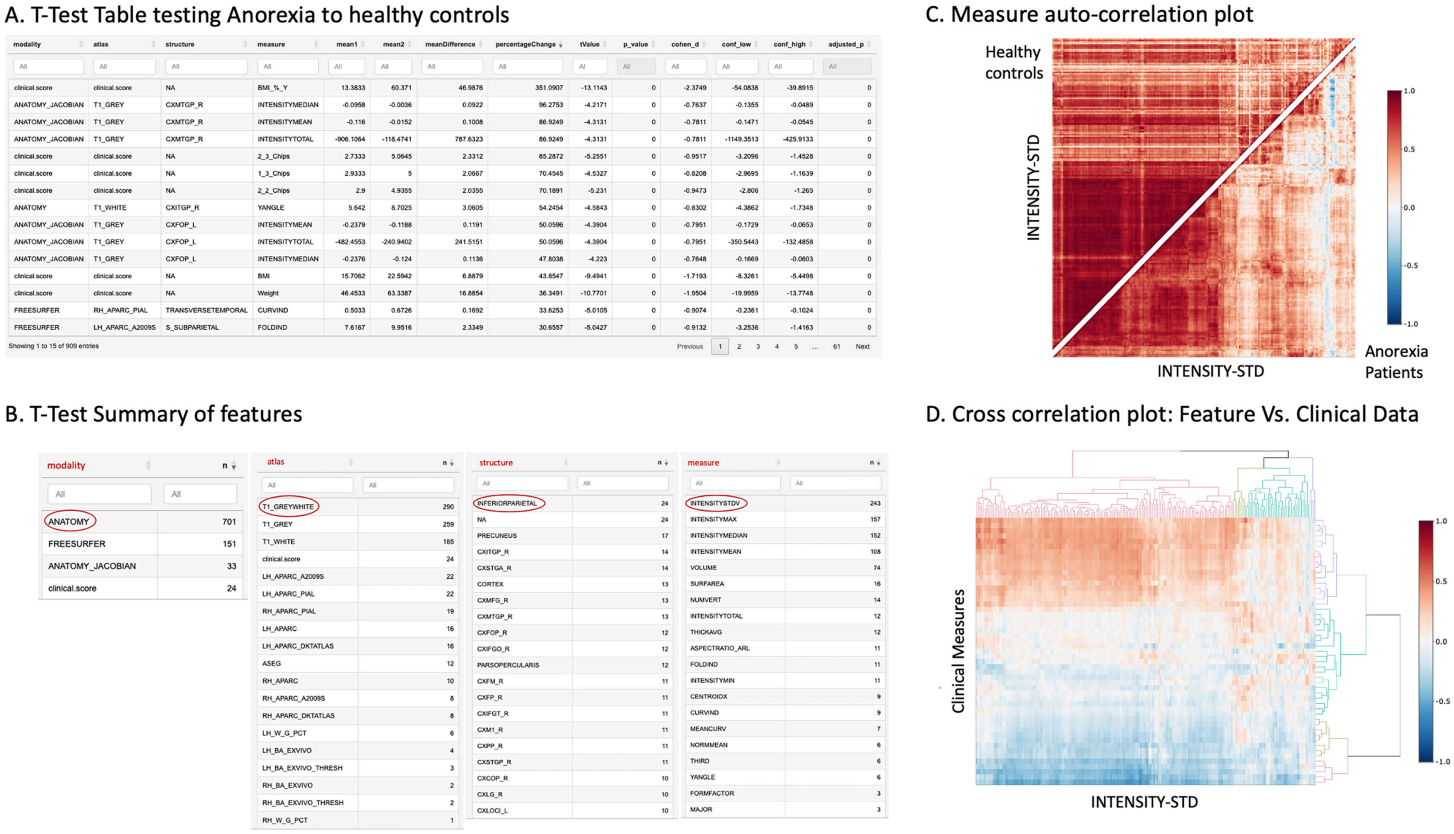
Traditional statistical analysis and correlation plots. **(A)** A detailed *t*-test table comparing specific brain measurements between anorexia and healthy control groups, with significant differences highlighted in various brain structures. **(B)** A summary of features tested, with the most prominent features (ANATOMY, T1_GRAYWHITE, INFERIOR PARIETAL, and INTENSITY-STD) highlighted in red ovals, which correspond to measures also utilized in Figure 3. **(C)** An autocorrelation plot with healthy controls in the top triangle and anorexia patients in the bottom triangle, demonstrating how ANATOMY T1 GW INTENSITY-STD, i.e., intensity standard deviation, correlates within each group. **(D)** A cross-correlation heatmap showing relationships between clinical scores and ANATOMY T1 GW INTENSITY-STD, further illustrating pattern differences between groups. This analysis builds upon the visualization in Figure 3 by quantifying the statistical significance of the observed group differences.

Figure 4 presents examples of advanced plots.

#### 2.6.2 Traditional statistical analysis

##### 2.6.2.1 Correlation analysis

MARIA provides tools to calculate and visualize correlation coefficients, which measure the strength and direction of relationships between variables. The tool presents correlation tables with *p*-values and supports multiple *p*-value correction options to ensure the robustness of the findings.

Figure 4C illustrates how these plots can be used to compare correlation patterns between different groups (e.g., anorexic patients vs. healthy controls). Figure 4D further shows how correlation analysis can be combined with hierarchical clustering to reveal patterns in complex datasets, for example, relationships between voxel intensity standard deviations across anatomical structures and clinical measures.

##### 2.6.2.2 *T*-test analysis

*T*-tests (paired and homo-/heteroscedastic unpaired, one- and two- sided) are employed to determine whether there are significant differences between the means of two groups. MARIA provides detailed output tables that include *t*-values, group-wise means, mean differences thereby indicating the direction of change, percentage changes, uncorrected *p*-values, and adjusted *p*-values. To control for multiple comparisons, users can select from several available *p*-value correction methods, including Bonferroni, Holm, Hochberg, Hommel, Benjamini-Hochberg (BH), Benjamini-Yekutieli (BY), and False Discovery Rate (FDR).

Figure 4A provides an example of such a table, showing the most significant features when comparing anorexic to the healthy control groups. This detailed output allows researchers to quickly identify which features show the most substantial group differences. Furthermore, as shown in Figure 4D, MARIA can summarize *t*-test results at different levels of data organization, such as by atlas, modality, or to specifics such as to the level of structures or measures providing a higher-level view of where significant differences are concentrated.

##### 2.6.2.3 Other analysis

MARIA also supports other traditional statistical methods for multi-group comparison methods such as ANOVA, ANCOVA, and MANOVA, which are not detailed here.

#### 2.6.3 Clustering and dimensionality reduction analysis

**FIGURE 5:**
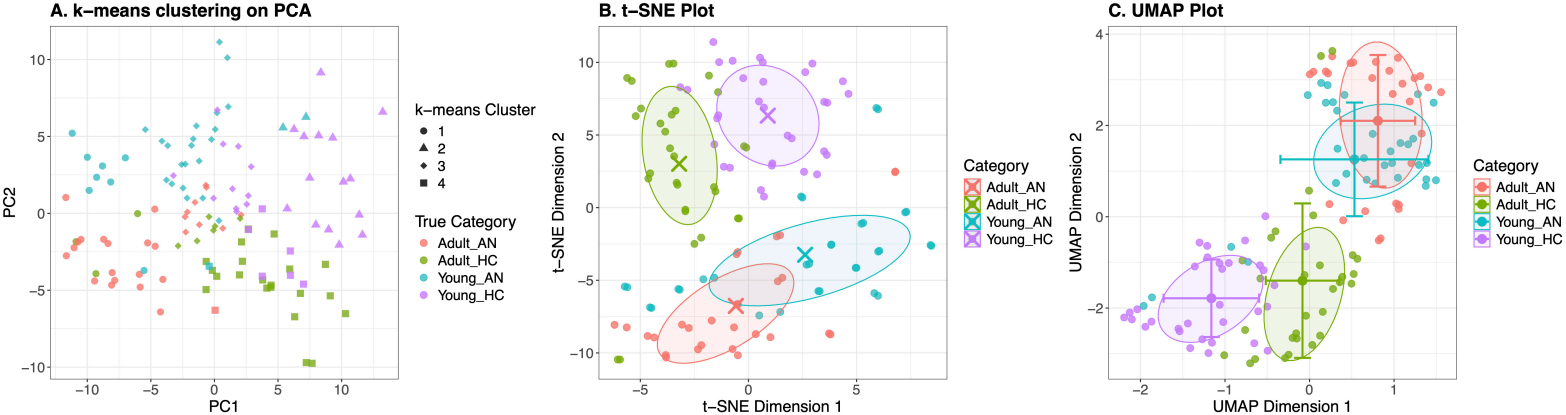
Dimensionality reduction plots. Clusters are clouded by study groups (Adult_AN, Adult_HC, Young_AN, Young_HC). **(A)** K-means clustering results on PCA components, comparing identified cluster patterns (shapes) with actual categories (colors). **(B)** t-SNE reduced dimensions, highlighting non-linear relationships between groups, along with centroids and ellipses for each category. **(C)** UMAP dimensional reduction, preserving both local and global data structures, while also presenting the standard deviation and ellipsoids. All visualizations were generated after feature selection using sPLS-DA. The analysis platform allows for flexible visualization options, including 2D/3D representations, custom coloring schemes (by measurement, center, or user-defined groups), various data scaling methods, and alternative feature selection approaches (supervised methods like sPLS-DA and Boruta, or unsupervised methods like PCA).

To explore patterns in high-dimensional data, MARIA offers several dimensionality reduction techniques to help researchers visualize and explore high-dimensional neuroimaging data. These techniques are useful to investigate, visualize group separation and identify complex, non-linear relationships. Figure 5 illustrates three popular dimensionality reduction methods implemented in MARIA: K-means clustering, t-SNE, and UMAP.

##### 2.6.3.1 Techniques supported

- Principal Component Analysis (PCA): PCA is used to reduce the dimensionality of the data by identifying the principal components that capture the most variance.
- Linear Discriminant Analysis (LDA): LDA finds linear combinations of features that best separate different classes.
- t-Distributed Stochastic Neighbor Embedding (t-SNE): t-SNE is a technique for reducing the dimensionality of high-dimensional data while preserving its structure, making it useful for visualization.
- Uniform Manifold Approximation and Projection (UMAP): UMAP is used to preserve both the global and local structure of high-dimensional data in a lower-dimensional space, aiding in the visualization and exploration of complex datasets.

##### 2.6.3.2 Visualization

As shown in Figure 5, MARIA provides interactive 2D and 3D scatter plots for various dimensionality reduction techniques. The dimensionality reduction techniques interactively allow for the creation of 2D or 3D scatter plots that represent high-dimensional data in a simplified form. These plots can be color-coded based on different variables or user-defined groups to visualize, but also identify separating parameters between clusters and patterns. Figure 5A illustrates how K-means clustering can be visualized in 2D, while Figures 5B,C show how t-SNE and UMAP results can be displayed in 2D and 3D, respectively. These visualizations allow researchers to explore potential separations between groups (such as anorexic vs. healthy groups for both adults and young subjects) and identify clusters or patterns in the data.

#### 2.6.4 Flexibility and user-friendly design

##### 2.6.4.1 External group information

MARIA allows users to incorporate external group information files to add and analyze new sub-groups or subsets of data. This feature enhances the flexibility and efficiency of data analysis by enabling dynamic group comparisons without reloading and processing the data.

##### 2.6.4.2 Publication-quality outputs

MARIA is equipped to generate and save publication-quality plots and tables from various statistical analyses. This functionality streamlines the process of integrating results into presentations or research papers, making it easier for researchers to present their findings.

### 2.7 ML Pipeline (machine learning pipeline)

The ML Pipeline is an automated and flexible R-framework designed to apply a comprehensive machine learning workflow to multi-parametric neuroimaging and clinical data. The entire process- from data preparation to model evaluation—is controlled by a single user-defined configuration file. This file allows researchers to specify all key parameters including:

- The data splitting strategy (e.g., training/test/ validation percentages).
- The cross-validation method (e.g., K-fold or Monte Carlo) and along with the number of iterations and number of folds.
- The specific feature selection and classification algorithms to be used.
- The random seed for ensuring reproducible results.

A sample configuration file, converted from YAML to human-readable PDF format for transparency, is provided in Supplementary material.

**FIGURE 6:**
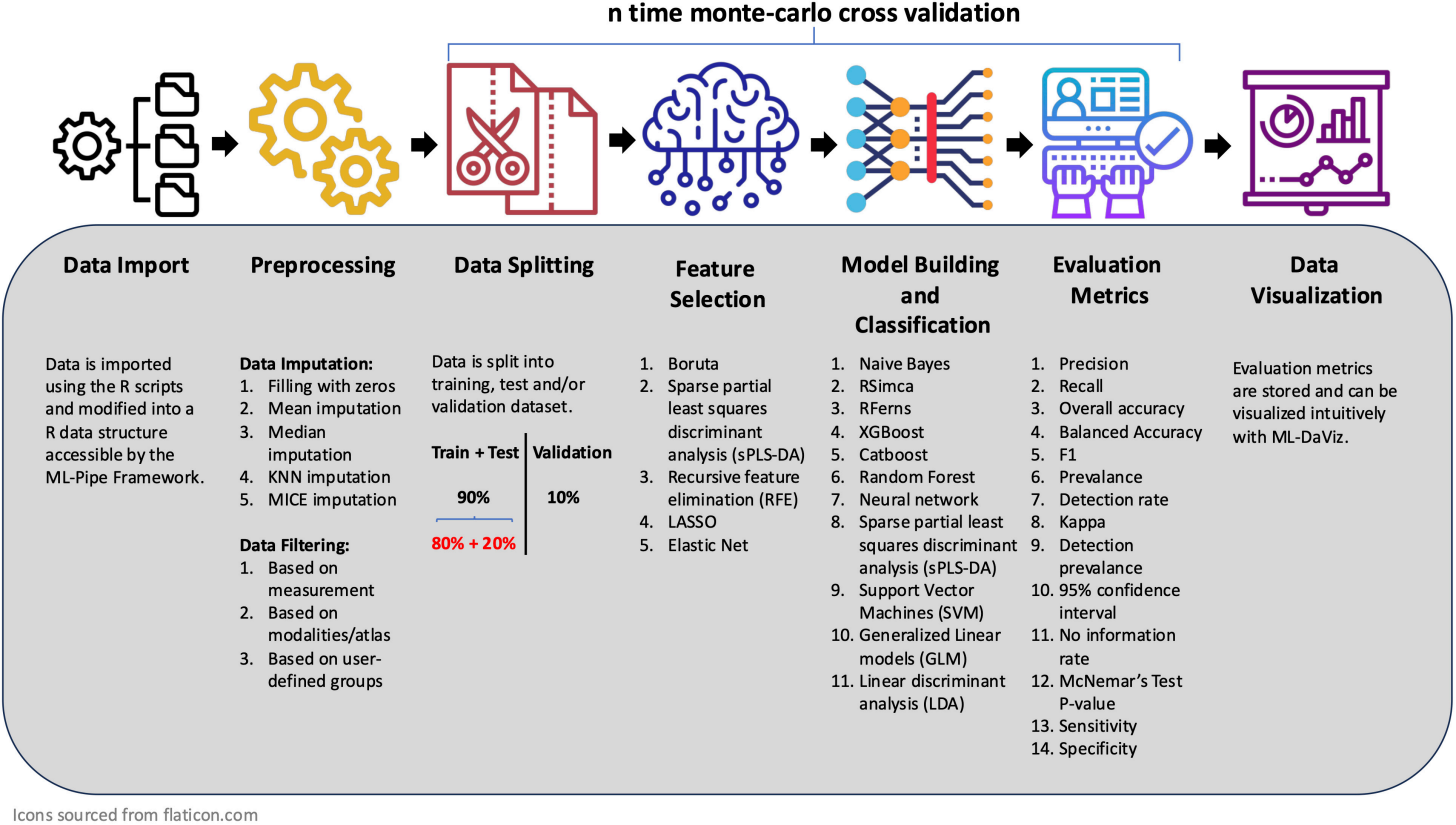
ML pipeline framework. This flowchart illustrates the automated and sequential workflow of the ML Pipeline, from initial data import to final model evaluation. Each stage is user-configurable via a central configuration file. All images were obtained from flaticon.com.

Once configured, the workflow runs automatically within a Singularity container, which encapsulates all necessary software packages and dependencies. This containerized approach ensures perfect reproducibility, platform independence, and allows the entire analysis to be seamlessly scaled on high-performance computing (HPC) clusters. The full workflow is depicted in Figure 6.

#### 2.7.1 Data Preparation and validation strategy

The initial phase involves thorough data preparation and pre-processing, which are crucial for ensuring the quality and relevance of the data used for model training.

- Data Filtering: An initial and vital filtering step allows users to define the precise dataset for analysis. Data can be a distinct subset based on analysis type (e.g., atlas-based), measurement time points, or user-defined groups specified in the external group file. Crucially, it also allows for the exclusion of specific features to prevent data leakage (e.g., removing features that directly encode the outcome variable).
- Data Splitting and Cross-Validation: To combat overfitting, validate metrics, and compensate for limited patient data in medical studies, ML Pipeline employs a robust cross-validation strategy. To ensure robust and unbiased model evaluation, the pipeline employs a nested validation strategy. First, the dataset is split into a training/testing set (e.g., 90%) and a final, held-out validation set (e.g., 10%). The validation set is left untouched until the final model evaluation. All subsequent steps, including scaling and tuning, are performed on the training/testing set using a stratified cross-validation strategy to preserve class balance. The pipeline offers two primary options: stratified k-fold cross-validation (default *k* = 5) and Monte Carlo cross-validation (repeated random sub-sampling).

#### 2.7.2 Feature engineering

Feature or variable selection is crucial for selecting a subset of relevant features (variables, predictors) from the high-dimensional data space. If the process is too conservative, the result is a noisy dataset, while being aggressive can result in discarding important information. Benefits of feature selection techniques include:

- Reduced training time
- Improved model interpretability
- Mitigate the curse of dimensionality
- Reduce the effect of overfitting

##### 2.7.2 1 Feature processing, selection

The ML Pipeline includes three different feature selection methods. Results can and should be cross-validated across all algorithms.

- Feature Scaling: To account for the diverse units and ranges in neuroimaging data, features are standardized using a Z-score normalization. This step is critical for distance-based algorithms like SVM and is performed within each CV fold to prevent information from the test fold from leaking into the training fold.
- *Boruta:* The Boruta algorithm (Kursa and Rudnicki, 2010) compares feature importance in the original dataset with the significance of shadow features created by random permutations. It typically utilizes a random forest classifier to assess feature importance. Features more important than their corresponding shadow features are retained. Boruta offers a robust and unbiased automatic feature selection process.
- *Sparse partial least square discriminant analysis (sPLS-DA):* The Sparse partial least square discriminant analysis (sPLS-DA) (Rohart et al., 2017) combines the principles of Partial Least Squares (PLS) regression (Cha, 1994) and LASSO (Least Absolute Shrinkage and Selection Operator) methods (Tibshirani, 1997). It performs a PLS decomposition and introduces a sparsity constraint to select a subset of features with strong discriminative power. This approach is particularly advantageous for high-dimensional data, as it reduces overfitting risk and improves classification model generalization by selecting a sparse feature subset.
- *Recursive Feature elimination:* Recursive Feature Elimination (RFE) (Chen and Jeong, 2007) is a technique that recursively fits a model and ranks the features based on their impact on performance. The least important features are eliminated iteratively until a desired subset is obtained. RFE helps improve model generalization, reduce overfitting, and enhance predictive accuracy by focusing on the most informative features, especially in high-dimensional datasets.

##### 2.7.2.2 Machine learning models: training and classification

The ML Pipeline supports a diverse suite of classification algorithms, allowing for a comprehensive comparison of different modeling approaches each with its unique strengths and applicability to different types of data and problem. The algorithms fall into several categories:

• Linear algorithms
  ∘ Generalized Linear Model (GLM): A binary logistic regression model that is easy to interpret but has strict assumptions about data distribution and sensitivity to outliers (Song et al., 2013).
  ∘ Linear Discriminant Analysis (LDA): Finds linear combinations of features to separate classes, reducing the curse of dimensionality but assuming normally distributed features (Tharwat et al., 2017).
  ∘ Naive Bayes: A simple probabilistic classifier based on Bayes theorem, effective in many real-world situations but missing dependencies between features (Rish, 2001).
  ∘ Support Vector Machine (SVM): Finds decision boundaries to separate classes, being memory efficient but sensitive to noise and less suitable for large datasets (Dimitriadou, 2009).
  ∘ Sparse Partial Least Squares Discriminant Analysis (sPLS-DA): Enables the selection of the most predictive or discriminative features to classify samples, quick but prone to overfitting (Rohart et al., 2017).
  ∘ Soft Independent Modeling of Class Analogy (SIMCA): A one-class classification method based on PCA, easy to add new classes but built independently (Filzmoser and Vlaskova, 2022).
• Tree-based methods
  ∘ Random Forest: An ensemble of decision trees that averages their predictions to improve overall performance, known for high-quality models but can be slow in prediction output (Liaw and Wiener, 2002).
  ∘ Extreme Gradient Boosting (XGBoost): Builds models sequentially to improve the previous model’s error, effective with large datasets but can overfit (Chen et al., 2019).
  ∘ CatBoost: A gradient boosting algorithm with categorical feature support, faster than XGBoost and effective for large datasets (Dorogush et al., 2018).
• Neural networks
  ∘ Neural Networks: Mimic the behavior of the brain with interconnected neurons, capable of handling extremely complex tasks but requiring significant power and training time, often difficult to interpret (Ripley et al., 2016).

We prioritized binary classification, i.e., two-class problems, because it directly addresses the most pressing clinical needs, such as diagnostic differentiation or predicting treatment response (e.g., responders vs. non-responders). Focusing on two-class problems allows the framework to generate more stable and interpretable “bio-signatures” for diagnostic decision-making. While the current framework is optimized for these tasks, the architecture is designed to eventually incorporate multi-class and regression models to track disease progression and continuous clinical scores.

##### 2.7.2.3 Hyperparameter tuning

- For each model, hyperparameter tuning is performed using a grid search methodology (Feurer and Hutter, 2019). This process is nested within the cross-validation loop; for each fold, the optimal combination of hyperparameters is identified based on the average performance across the training partitions.

#### 2.7.3 Model evaluation and output

Models are trained in parallel, and their performance is rigorously evaluated. For each model and feature selection combination, the pipeline calculates a comprehensive set of performance metrics (e.g., accuracy, balanced accuracy, sensitivity, specificity, precision) on the training, test, and held-out validation sets. All results, including class predictions, probabilities, and the list of selected features for each iteration, are saved as .RDS files, ready for visualization and interpretation in the ML DaViz framework.

### 2.8 ML DaViz (machine learning analysis data visualization)

ML DaViz is an intuitive GUI tool developed in R using the shiny package to support detailed analysis and visualization of results from the ML Pipeline. It allows researchers to comprehensively evaluate model performance, interpret feature importance, and visualize complex relationships within neuroimaging data. A detailed explanation of individual scores and metrics is beyond the scope of this paper.

#### 2.8.1 Overview and capabilities

ML DaViz serves as the visualization and analysis component of the BrainInsights framework, specifically focusing on the outcomes generated by the machine learning models in ML Pipeline. Designed for ease of use, it allows researchers at all levels to interact and interpret their ML results effectively. With the different visualization techniques and metrics provided the user can also compare different analysis levels like modalities and atlases with their performance in different algorithms at levels of feature selection as well as feature classification.

#### 2.8.2 Model performance analysis

**FIGURE 7:**
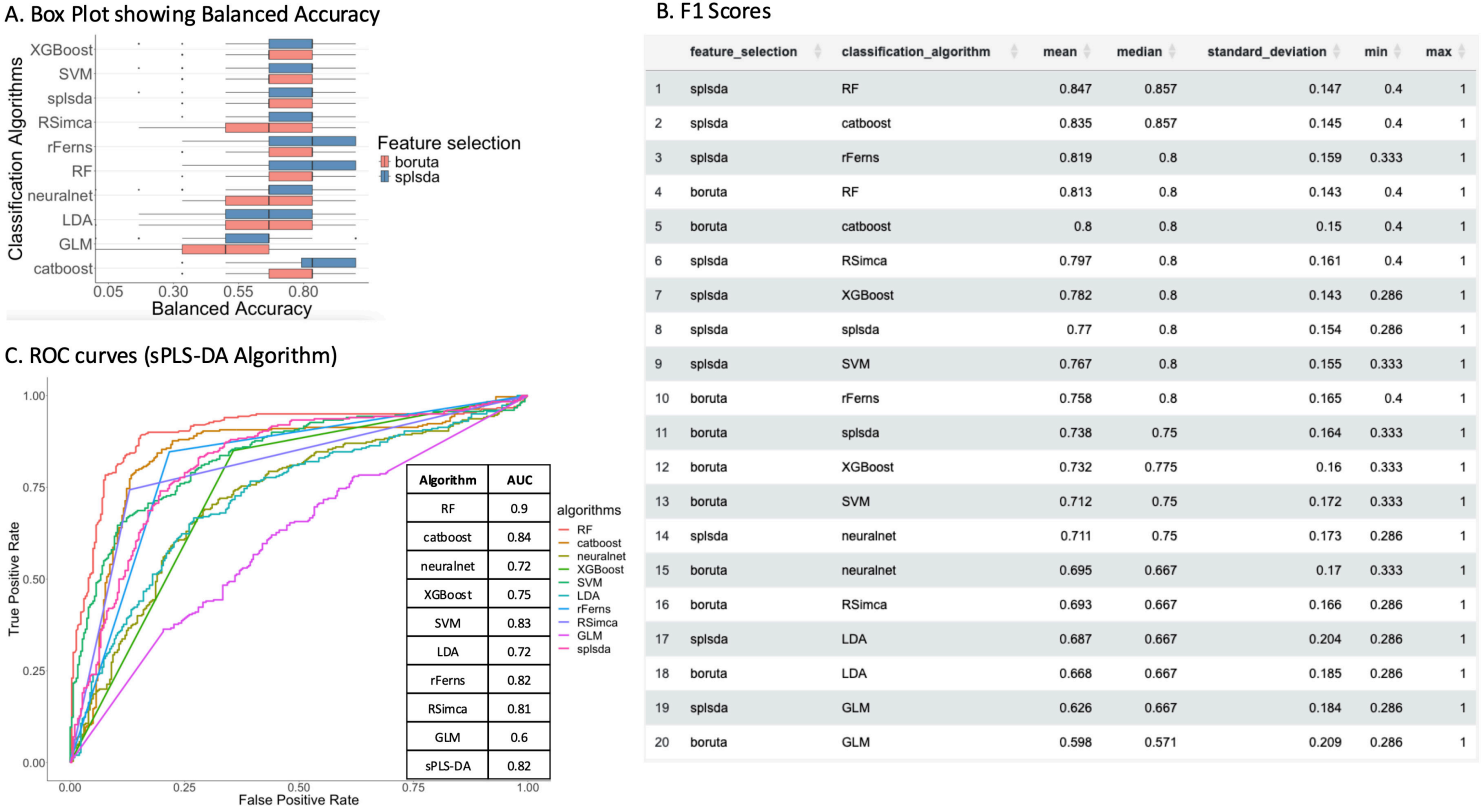
Metrics from Anorexia Young sample are presented in the figures. **(A)** A Box plot illustrating the distribution of Balanced Accuracy scores achieved by different classification algorithms (listed on the y-axis). Within each algorithm, performance is compared based on prior feature selection: results using Boruta feature selection are shown in red, and results using sPLS-DA feature selection are shown in blue. Several algorithms demonstrate high median accuracy, particularly when paired with sPLS-DA features. **(B)** A table summarizing the F1 scores obtained for the various combinations of classification algorithms and feature selection methods evaluated. **(C)** A plot with Receiver Operating Characteristic (ROC) curves comparing the performance of different classification algorithms when applied after feature selection using the sPLS-DA method. The corresponding Area Under the Curve (AUC) values are provided at right side, quantifying the discriminative ability of each model combination.

- Confusion Matrix Metrics: Visualize confusion matrix metrics as tables, heatmaps, violin plots, and histograms to assess accuracy, precision, recall, and F1-score.
- ROC Curves: Interactive ROC curves with AUC values to evaluate and compare the diagnostic performance of binary classifiers (Figure 7).
- Other Metrics: View additional performance metrics such as accuracy, balanced accuracy, precision, recall, and F1-score.

#### 2.8.3 Feature selection distribution analysis

**FIGURE 8:**
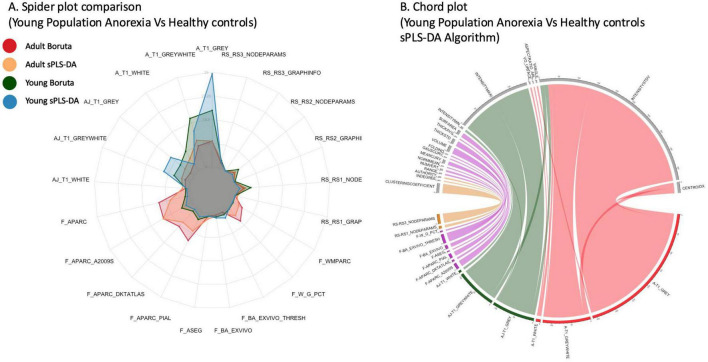
Feature distribution and selection stability. **(A)** A spider plot comparing features selection frequency (the percentage of 100 Monte Carlo iterations in which a feature was selected) by sPLS-DA and Boruta algorithms across adult and young cohorts. Each axis represents a specific brain atlas, with clouded regions indicating selection frequency for each algorithm-subgroup combination (Adult-Boruta, Adult-sPLS-DA, Young-Boruta, Young-sPLS-DA) and the distance from the center indicating higher selection stability (0–100%). **(B)** A chord plot specifically for the sPLS-DA Algorithm for the young subjects group, visualizing the relationships between selected features, different brain atlases, and measurement types. The thickness of the connecting links represents the relative strength of the relationship between selected features, while node colors distinguish between different MRI modalities, atlases and specific brain measures. These visualizations allow for comparative assessment of feature selection stability across different classification approaches and age groups, highlighting the most consistent neuroanatomical markers of anorexia.

A key goal of ML DaViz is to move beyond model performance and identify the features that consistently drive classification. The feature selection distribution analysis is designed to assess the biological relevance and stability of these features. This analysis reveals which modalities, atlases or specific brain measures are most frequently selected across different algorithms and cross-validation iterations, while also allowing for evaluation of the selection process’s stability. To facilitate this, ML DaViz provides visualizations as shown in Figure 8.

Spider plots are used to directly compare the selection frequency of features across different algorithms or subgroups.

Chord plots offer a detailed view of relationships between the most important features and their corresponding atlases and measures. Together, these tools are essential for interpreting the model’s findings and building a robust, data-driven bio-signature.

#### 2.8.4 Model interpretability and bio-signature creation

Moving on from creating models that can classify accurately, a more essential task is understanding what makes the model perform. The shift from predictive accuracy to explanatory insights is crucial for several reasons. It allows to move beyond a black-box approach toward an interpretable model approach, building necessary trust for clinical transition. Understanding what goes on inside the model, helps us generate new hypotheses about underlying biology. Furthermore, it can also be used to identify if the prominent features arise due to technical artifacts, serving as a crucial quality control step.

The process of understanding and explaining a model’s behavior is known as model interpretability. It can be approached at two levels: global interpretability, which explains the model’s overall behavior (e.g., which features are generally most important), and local interpretability, which explains the reason behind a prediction for a single subject. By incorporating these additional explainability to the black-box models, ML DaViz bridges the gap and aids toward a robust bio-signature creation.

Model Interpretability Techniques: To provide deeper insights, the framework has been incorporated with these state-of-the-art techniques.

- Mean Decrease Accuracy
- LIME (Ribeiro et al., 2016) (Local Interpretable Model-agnostic Explanations)
- SHAP (Lundberg and Lee, 2017) (Shapley Additive exPlanations)

Bio-signature creation: The creation of a bio-signature is the synthesis of these feature selection and interpretability analyses. It is more than just selecting the top selected features, but to identify a stable pattern that is biologically plausible. The process within BrainInsights is a two-stage workflow designed to build a single, robust and interpretable model from the aggregated results of the ML Pipeline.

1. Identify Stable Features: Using ML-DaViz selected features from the different feature selection algorithms are aggregated across n iterations. A feature is considered stable if it was consistently selected across the runs (e.g., > 30% of the iterations). This threshold was chosen as a heuristic to balance the inclusion of informative markers with the exclusion of noisy predictors that may appear sporadically due to sampling variability or high collinearity in small clinical cohorts.
2. Understand Feature Contributions: Second, a final interpretable model is trained using only this stable feature set. The framework then employs interpretability techniques like SHAP to explain this final model. This analysis quantifies how each stable feature and its interactions contribute to the prediction (e.g., its direction and magnitude), moving the model from a black-box to an interpretable tool.
3. Synthesize the Multi-modal signature: A robust bio-signature is not often a single feature or a marker but a collective profile derived from the most influential features. The framework’s flexibility aids in building a signature that is comprised not only of features from multi-modal MRI data but also clinical data, that represents a reproducible fingerprint for a specific condition, cohort or treatment outcome. The two-step process ensures that the resulting bio-signature is not only reproducible across different data splits but also comprises the most influential drivers of the classification outcome.

**FIGURE 9:**
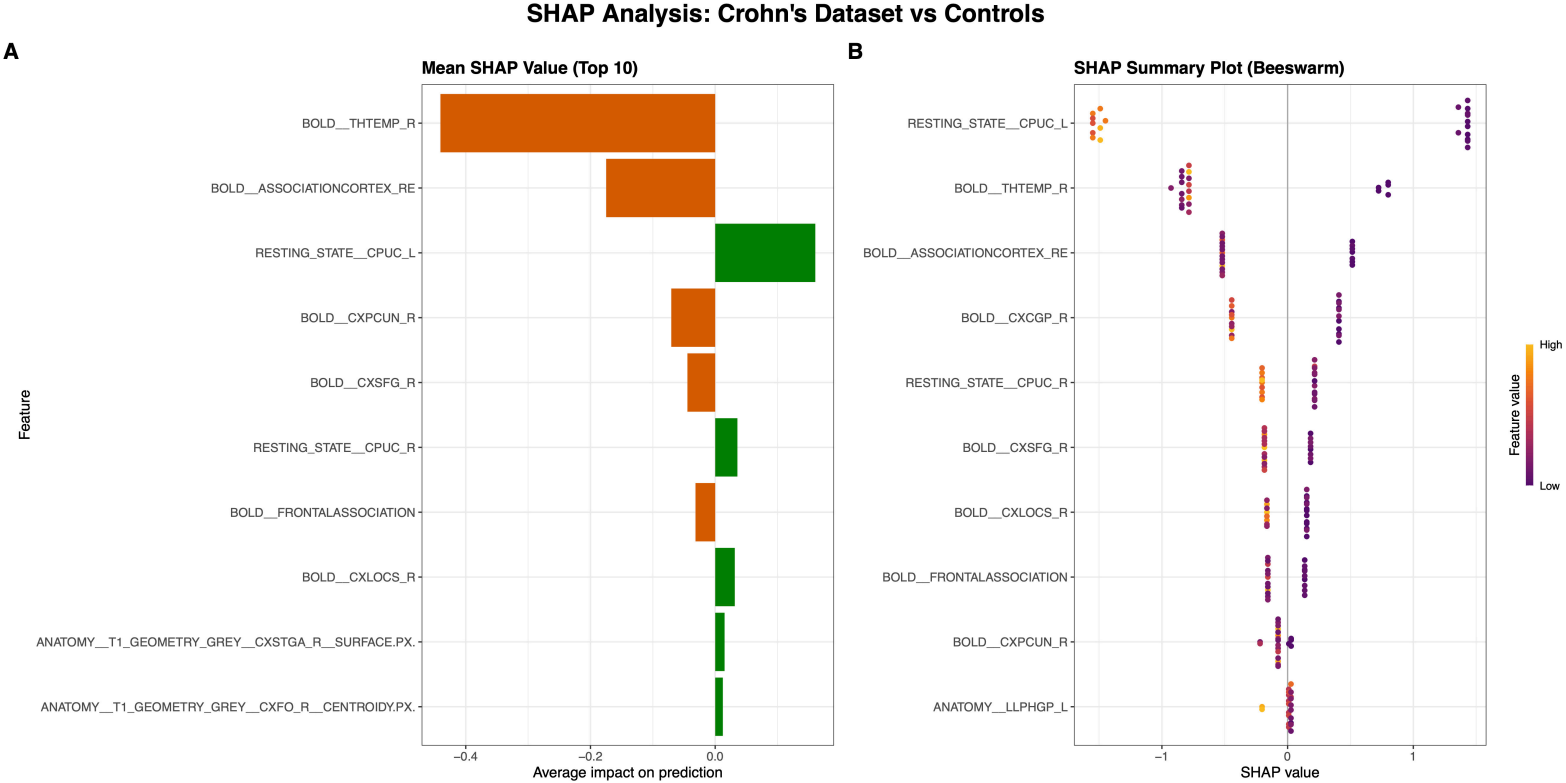
Model Interpretability using SHAP plots in ML DaViz. These plots explain a model trained to differentiate Crohn’s disease patients from healthy controls, evaluated on a held-out test dataset. **(A)** The Mean SHAP Value of the top 10 features, that shows the impact of the most important features on the model’s prediction. Orange bars (e.g., BOLD_THTEMP_R) push the prediction toward one of the classes (in this case controls) while green bars (e.g., RESTING_STATE_CPUC_L) push them toward the other class (Crohn’s disease in this case). **(B)** The SHAP summary plot, showing the distribution of SHAP values for each test subject. The color indicates the feature’s value (High = Yellow and Low = Purple), revealing how a feature’s value relates to its impact on prediction.

A practical example of this interpretability analysis is shown in Figure 9, which uses SHAP plots generated by ML DaViz to explain a model trained to differentiate patients with Crohn’s disease from healthy controls and the results are presented from a held-out unseen test dataset.

The Mean SHAP value plot (Figure 9A) summarizes the global model behavior, ranking the top 10 features by average impact. Features with a positive mean SHAP value, like RESTING_STATE__CPUC_L (green bar), generally drives the prediction toward the “Crohn’s disease” while features with negative mean values (orange bars), drive the prediction toward the control data.

The SHAP summary plot (Figure 9B) provides a more granular, local-level explanation by visualizing the distribution of SHAP value for every individual subject for the top features that drive the prediction of the model. This plot details both the magnitude of a feature’s impact and its direction based on the feature’s original value.

- High feature values (yellow/red dots) correspond to high positive SHAP values, strongly associating them with a “Crohn’s disease” prediction.
- Low feature values (purple dots) result in larger negative SHAP values, driving the prediction toward “Healthy controls.”

This ability to drill down to subject-specific insights is what transforms the model from a black-box into a interpretable one. By identifying stable biologically plausible drivers, ML DaViz provides the necessary foundation toward building a robust bio-signature.

#### 2.8.5 Model comparison

ML DaViz facilitates the comparison of multiple machine-learning models, helping researchers to identify the most effective models for their data.

• Performance metrics comparison
  ∘ Side-by-Side Comparison: Researchers can compare key performance metrics (e.g., accuracy, AUC, precision, recall) of different models side-by-side.
  ∘ Statistical Significance: The tool can also provide statistical tests to determine if differences in performance metrics between models are significant.
• Feature importance comparison
  ∘ Visual Comparison: Visual tools like bar charts and heatmaps enable the comparison of feature importance across different models, providing insights into which features are consistently important.
  ∘ Consistency Analysis: By analyzing the consistency of feature selection across different models, researchers can gain confidence in the robustness of their findings.

#### 2.8.6 Exporting and reporting

To facilitate inclusion in publications and presentations, all tables and visualizations can be easily exported. Visualizations are available in high-resolution formats like PNG and PDF, while data tables can be saved as CSV or Excel files. ML DaViz also includes a feature to generate comprehensive summary reports of the analysis.

## 3 Case studies

To showcase the capabilities and integrated approach of BrainInsights, we present three different case studies. We conducted extensive experiments using several datasets. A summary of the dataset sizes, modalities used and primary objectives for the case studies is provided in Supplementary Table 4. Moreover, in those publications, the results obtained by machine learning and dimensionality reduction showed a very good cross-validation with traditional statistics. These examples highlight how the framework addresses challenges across different research scenarios, from initial data exploration and hypotheses generation to advanced machine learning models and systematic errors detection.

### 3.1 Case study 1: standard workflow

This case study showcases a typical BrainInsights workflow using data from a Neurotrition study comparing adolescents and adults with Anorexia Nervosa (AN) to age-matched healthy controls (HC). Detailed information regarding the study design and participant demographics can be found in the work published by Mendez-Torrijos et al. (2024). The analysis demonstrates the full sequence of data importation, exploratory analysis, statistical testing, machine learning, and results interpretation.

#### 3.1.1 Step 1: data import and curation (MARIA)

First, we imported the raw neuroimaging features (csv/sylk) and clinical data (xlsx) into R data frames using the available scripts. We then applied the data curation pipeline which checked for data consistency, naming conventions, duplicates and excluded features with over 30% missing values, to avoid introducing potential bias and noise. For the remaining missing data we used the Multivariate Imputation by Chained Equations (MICE) algorithm on an atlas-based level resulting in a complete, structured dataset ready for analysis. Though time and computationally intensive, MICE effectively handled the dataset’s complexity.

#### 3.1.2 Step 2: data inspection and statistical exploration (MARIA)

The curated data was set was examined using MARIA visualization tools to identify trends or potential issues. Traditional statistical analyses were then conducted.

##### 3.1.2.1 *T*-test analysis

To identify significant group differences between AN and HC groups, we performed a *t*-test analysis. This revealed features showing substantial differences between the groups, with detailed results including *t*-values, group means, mean differences, percentage changes, and raw and adjusted *p*-values available (Figure 4A). Summary tables (Figure 4D) further aggregated these findings by modality, atlas, structure, or measures, highlighting areas where differences were most concentrated.

##### 3.1.2.2 Correlation analysis

We then investigated the relationships between these significant features and clinical variables using correlation plots. Auto-correlation plots (Figure 4C) revealed relationships within an atlas, while cross-correlation plots (Figure 4D) demonstrated associations with clinical variables.

##### 3.1.2.3 Results

The Anatomy T1 Gray White atlas and standard deviation of intensity, identified as dominant by the *t*-test summary table (Figure 4B), were utilized in the plot to compare differences. For example, Figure 4D depicted how these features correlated differently with various clinical parameters.

#### 3.1.3 Step 3: feature visualization based on statistical insights (MARIA)

Building on insights from the statistical analysis, selected features of interest were visualized using the different plotting options available in MARIA.

##### 3.1.3.1 Line plots and density plots

Figures 3A,B show the inferior parietal volume, identified as one of the most significant features in the *t*-test results (Figure 4A), using a line plot and density plot. The Line plot shows changes in volume between the sub-groups from measurement one to two. Figure 3B, illustrates how the inferior parietal volume was spread for the sub populations before treatment, with dotted lines indicating the mean volume for comparison.

#### 3.1.4 Step 4: exploratory group separation analysis (MARIA)

Following the individual feature analysis, we tested our hypotheses with the help of dimensionality reduction algorithms like k-means clustering, tSNE and UMAP after the features were selected using sPLS-DA algorithm as shown in Figure 5. The visualizations showed group separation across the algorithms and this provided us with confidence to continue further with the more time consuming and computationally intensive machine learning model building and validation.

#### 3.1.5 Step 5: machine learning model construction (ML Pipeline)

The insights gained from statistical analysis and hypotheses testing in MARIA were used in the user-defined configuration file. The pre-processed data from the import step was subsequently provided to the ML Pipeline for building the machine learning models. The automated pipeline normalized the data using z-score normalization and performed hyperparameter tuning. The data was then split using stratified sampling to balance the classes (75% training and 25% testing) based on a random seed. Feature selection was conducted using either the sPLS-DA or Boruta algorithm. Subsequently for each feature set, all machine learning models listed in Figure 6 were constructed for each feature set. These steps were repeated 100 times using monte-carlo cross-validation to ensure a reliable evaluation of model performance and stability.

#### 3.1.6 Step 6: model evaluation and bio-signature generation (ML DaViz)

The ML models built using ML Pipeline were evaluated using the ML DaViz application, which is crucial for interpreting model performance and generating bio-signatures.

##### 3.1.6.1 Performance metrics and ROC curves

Analysis of model performance revealed distinct patterns across different classification and feature selection approaches. For instance, balanced accuracy distributions (Figure 7A) indicated that certain classifiers, particularly when paired with sPLS-DA feature selection, consistently achieved higher and more stable performance across iterations. F1 scores for young subjects separating AN from HC (Figure 7B) further corroborated these findings, highlighting effective model combinations. Receiver Operating Characteristic (ROC) curves and corresponding Area Under the Curve (AUC) values (Figure 7C) provided a visual assessment of the discriminative power of different algorithms, demonstrating their capacity to differentiate between groups.

##### 3.1.6.2 Feature selection distribution analysis

To understand the feature contribution most to classification, ML DaViz presents us with spider plots and chord plots. These visualizations offered insights into the stability and consistency of feature selection across different algorithms and subject groups. For example, Figure 8A, a spider plot for adult and young subjects, allowed for direct comparison of features consistently selected by Boruta and sPLS-DA algorithms over iterations at an atlas level. Options exist to compare at a modality-based level or a more specific measure-based level for a given modality or atlas. Figure 8B, a chord plot, further detailed the distribution of features selected by the sPLS-DA algorithm for the young subjects group, visualizing the intricate relationships between modalities, parametric MRI, atlases, and specific measures, thereby illustrating which features collectively contributed to group discrimination.

Finally, to complete the workflow, we applied the interpretability tools described in ML DaViz section. This crucial step transforms the model from black-box into an interpretable one. As shown in our analysis of the Crohn’s disease dataset (Hess et al., 2015; Figure 9), stable features are identified, that are biologically plausible drivers, which forms the basis of a robust bio-signature.

### 3.2 Case study 2: dimensionality reduction as a hypotheses testing tool

This case study demonstrates in more detail how MARIA’s dimensionality reduction tools can be used for data-driven hypothesis generation. We showcase a hierarchical “drill-down” analysis to interactively identify the most discriminative neuroimaging modalities and measures from a complex dataset.

The visual separation was validated by machine learning models built using the specific data subsets in ML Pipeline, with performance metrics strongly corroborating the visual separation.

**FIGURE 10:**
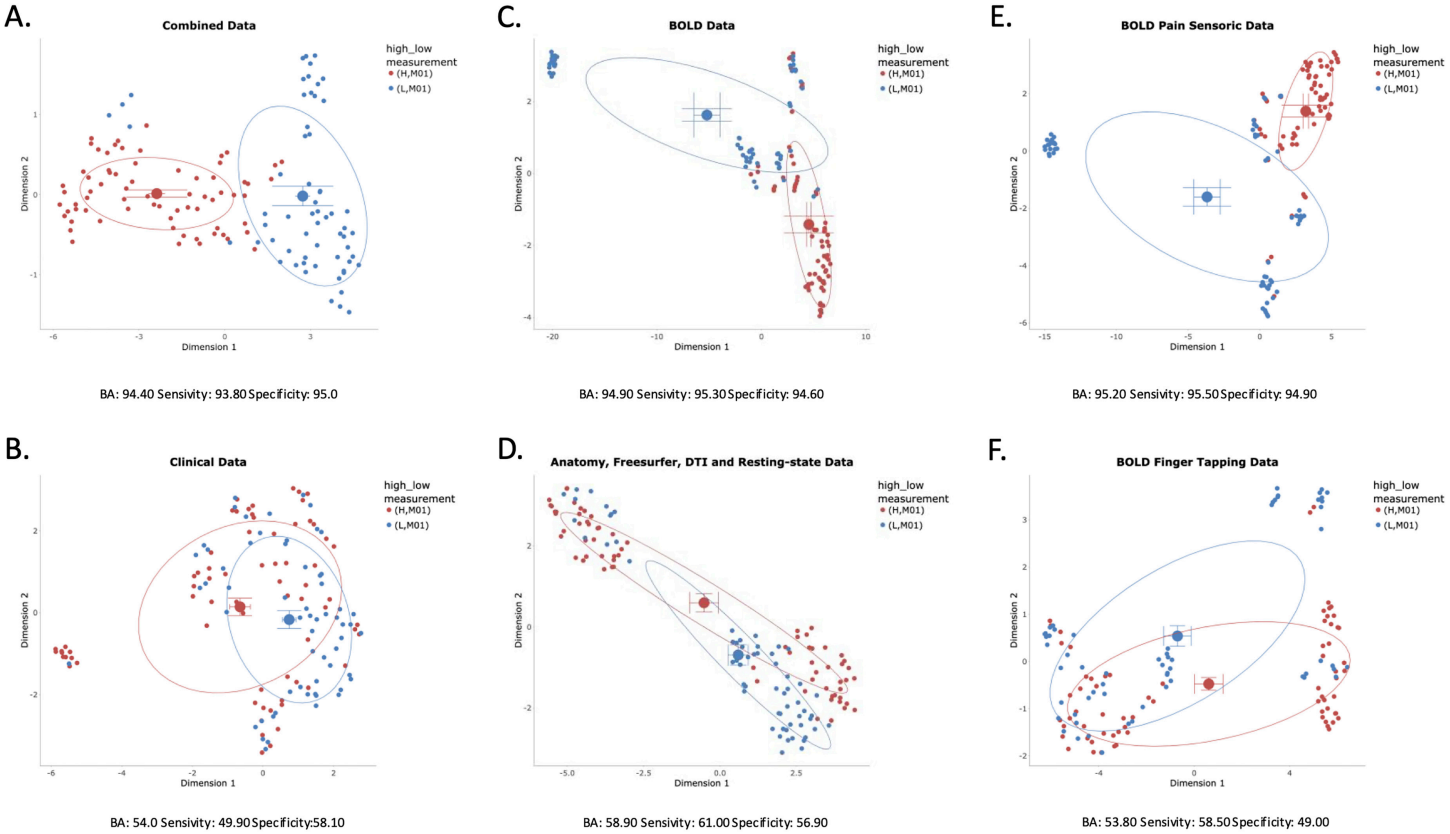
Hierarchical dimensionality reduction for hypotheses testing. UMAP plots showcasing group separation between high and low voxel activity groups (H_M01 and L_M01) at baseline (M01) from the PreCePRA trial. All visualizations were generated after feature selection using sPLS-DA. **(A)** Illustrates good visual separation with combined clinical and multi-parametric MRI data, yielding strong machine learning model performance [Balanced Accuracy (BA): 94.40%, Sensitivity: 93.80%, Specificity: 95.00%]. In contrast, **(B)** that clinical data alone results in mostly overlapped clusters and poor model performance (BA: 54%), indicating that traditional clinical measures were not the primary drivers of differences. **(C)** Highlights that among individual parametric-MRI analyses, only the BOLD dataset achieved clear visual separability, which was supported by promising model performance (BA: 94.90%). Conversely, **(D)** demonstrates no visual separation when combining multi-parametric MRI features without BOLD data, leading to limited machine learning discrimination (BA: 53.80%). Further breakdown within the BOLD subset reveals that pain-related brain activity **(E)** achieved the best visual and machine learning separation (BA: 95.20%), while finger tapping data **(F)** performed much worse (BA: 58.90%).

For this analysis, we used data from the PreCePRA trial, phase 3 trial on treatment of rheumatoid arthritis patients by Certolizumab, an anti-TNF biological (for study details please see Hess et al., 2025). The patients stratified into “high” and “low” groups based on their baseline fMRI-measured CNS pain activation and the hypothesis was, that “high” voxel responders are more likely to benefit from the anti-TNF therapy. As shown in Figure 10, we here used UMAP to visualize group separability after performing feature selection with sPLS-DA. By systematically analyzing different data subsets, we pinpointed the precise data driving the separation, validating each visual finding with quantitative performance from machine learning models built in the ML Pipeline.

#### 3.2.1 Our hierarchical investigation proceeded as follows

- 1. Full Dataset Analysis: We first analyzed the complete, combined dataset (multi-parametric MRI and clinical data). The UMAP plot revealed a clear visual separation between the high and low activation groups, which was confirmed by strong classification performance (e.g., Balanced Accuracy > 85%).
- 2. Clinical Data Alone: Next, to test the value of neuroimaging, we analyzed the clinical data by itself. The UMAP plot showed significant cluster overlap, and the corresponding ML model performance was no better than chance (e.g., Balanced Accuracy ≈ 50%). This demonstrated that clinical data alone was insufficient to distinguish the groups.
- 3. Isolating MRI Modalities: We then investigated which imaging modality was responsible for the separation. After analyzing each parametric MRI dataset individually, we found that only the BOLD fMRI data produced clear visual separability and high classification accuracy. When we explicitly excluded the BOLD data from the full multi-parametric set, the separation disappeared.
- 4. Pinpointing the BOLD Signal: Finally, we dissected the BOLD data further. The analysis revealed that pain-related brain activity achieved excellent group separation, while activity from a control “finger tapping” task performed poorly.

This hierarchical approach allowed us to move beyond a simple “black-box” classification and identify pain-related functional imaging as the key discriminative feature set. This finding not only validates the BrainInsights workflow but also suggests specific future directions for understanding group-specific pain processing networks.

### 3.3 Case study 3: leveraging MARIA for data inspection and quality control

This case study highlights MARIA’s critical role in quality control and troubleshooting. It demonstrates how the ability to move iteratively between machine learning outputs and data inspection is essential for validating results and uncovering the story behind the data. We present two real-world scenarios: (1) identifying a technical artifact and (2) validating an unexpected biological finding.

#### 3.3.1 Initial data quality assessment

Before conducting advanced analysis, MARIA’s visual tools help detect outliers or inconsistent data distributions. Simple plots such as histograms and density plots, enable rapid inspection and correction.

#### 3.3.2 Example 1: retrospective error identification—the “100% separation” anomaly

A common red flag in machine learning is a model that achieves perfect (100%) or near-perfect group separation. While seemingly ideal, this often points to data leakage or a systematic error rather than a true biological discovery.

- *Scenario:* In one analysis comparing actual patient data to an older control dataset, our ML Pipeline reported a puzzling 100% classification accuracy. This was highly suspect, especially since a standard VBM analysis on the same data showed no clear group differences.
- *Investigating with MARIA:* To resolve the discrepancy, MARIA’s data inspection capabilities became essential. Analysis of the top features selected for separation using density plots revealed a consistent scaled difference between the groups.
- *Identifying the root cause:* The pattern appeared too clean to be biological. A closer look at the data processing logs revealed the issue: the datasets had been processed by different researchers with inconsistent parameterization using the software MagnAn V2.6 (BioCom GbR, 2026). This discrepancy in software settings introduced a systematic, non-biological artifact across many features, which the machine learning algorithm correctly exploited to achieve perfect, but misleading, separation. MARIA’s visualization capabilities were crucial for quickly diagnosing this technical error and preventing a misleading conclusion.

#### 3.3.3 Example 2: validating a “surprising” biological finding

In our Neurotrition study analysis, we encountered an unexpected result, at least for us: adolescents with AN appeared to have *higher* total gray matter volume than healthy adults.

- Initial Observation: We first observed this trend in MARIA’s boxplots (Figures 3C,D). While surprising, the pattern was clear.
- Cross-Method Validation: To ensure this was not an artifact of a single method, we cross-referenced the finding with results from our VBM pipeline. The VBM analysis independently confirmed the same pattern of higher gray matter volume in the adolescent group, strengthening our confidence in the initial observation.
- Assessing Biological Plausibility: This consistent finding prompted a literature review for a biological explanation. Indeed, this observation aligns with current knowledge of adolescent brain development. The brain undergoes a dynamic phase of synaptic pruning during adolescence, where initially greater gray matter volume is refined to enhance neural efficiency (Blakemore and Choudhury, 2006; Gogtay et al., 2004). What appeared to be an anomaly to us at first hand was, in fact, a reflection of a known neurodevelopmental process.

These examples underscore the importance of treating data analysis not as a linear pipeline, but as an iterative cycle of discovery and validation. BrainInsights is designed to facilitate this critical process, enabling researchers to distinguish technical artifacts from genuine—and sometimes surprising—biological insights.

## 4 Discussion

First, we summarize the principal findings and advancements provided by BrainInsights. Next we pinpoint the strengths of BrainInsights by comparing it to existing frameworks and end by discussing actual limitations and provide future directions.

### 4.1 BrainInsights as an integrated ecosystem

The principal novelty of BrainInsights lies not in any single feature, but in its design as a holistic and integrated ecosystem. This ecosystem lowers the technical barrier for neuroimaging research by offering both a user-friendly GUI for interactive exploration and automated scripts for batch processing. However, its true strength emerges from the seamless integration of its components. This allows researchers to follow a complete chain of reasoning: from initial data exploration and hypothesis generation in MARIA, to robust model building in the ML Pipeline, and finally to deep interpretation of results in ML DaViz.

### 4.2 Bridging the gap between statistics and machine learning

A core contribution of BrainInsights is its deliberate design to bridge the gap between two complementary scientific paradigms: traditional hypothesis-driven testing and modern data-driven discovery. Our framework treats them not as competing approaches, but as synergistic stages of a single investigative process, allowing researchers to move fluidly between them.

The power of this integration is twofold. First, it allows statistical inference to directly inform machine learning. As demonstrated in Case Study 1, we began with a hypothesis-driven approach, using traditional *T*-tests in MARIA to identify statistically significant features. This provided a transparent, data-grounded rationale for the subsequent ML analysis, ensuring that our predictive models were built upon interpretable biological differences rather than being treated as a “black-box.”

Second, the framework supports the reverse workflow, where machine learning drives hypothesis generation. In Case Study 2, we took an open-ended, data-driven approach. By using dimensionality reduction and classification models to ask “what features, if any, separate these groups?,” we discovered that specific fMRI measures were the key differentiators. This data-driven finding generated a new, highly specific hypothesis about the role of pain-processing networks that can now be tested in future studies. By uniting the *inferential* power of statistics with the *exploratory* power of machine learning, BrainInsights creates a dynamic workflow that mirrors the real cycle of scientific inquiry.

### 4.3 Fostering robustness and reproducibility through iterative analysis

Perhaps the most significant advance offered by BrainInsights is its support for a non-linear, iterative cycle of analysis and validation. Modern computational research can often feel like a linear “pipeline,” where data goes in one end and a result comes out the other, with little room for introspection. Our framework is designed to break this paradigm.

As powerfully demonstrated in Case Study 3, this iterative capability is critical for ensuring scientific robustness. When the ML Pipeline produced a “too good to be true” 100% accuracy, the ability to immediately take those top features back into MARIA for visual inspection was not just convenient; it was essential. It allowed us to diagnose the issue as a technical artifact stemming from an inconsistent software setting during the initial feature extraction phase in MagnAn, thereby preventing the publication of a spurious finding.

Conversely, this same iterative loop provides a mechanism for building confidence in surprising *and real* biological findings. The unexpected observation of higher gray matter volume in adolescents was initially met with skepticism, but its validation across different analytical methods within the framework gave us the confidence to explore its plausible biological basis in synaptic pruning. By enabling researchers to fluidly move between predictive modeling, statistical testing, and visual exploration, BrainInsights transforms potential errors into learning opportunities and ensures that final conclusions are both statistically sound and scientifically plausible. Finally, it is this rigorous, iterative validation that provides the necessary confidence to move beyond simple classification and begin carving out robust, disease-specific or treatment-specific bio-signatures.

This focus on interpretability is central to this framework. The integration of SHAP to identify predictive drivers is only a first step. For a truly deep understanding, required for a single-subject clinical translation, efforts are required to move beyond feature importance to understand complex feature interactions and causal pathways. This remains a significant and complex challenge in this field.

### 4.4 Strengths and advantages over existing tools

When compared to existing frameworks, BrainInsights offers several key advantages in both design and practical implementation. Many established tools require significant programing expertise, creating a barrier for researchers focused on clinical and neuroscience questions. By providing a powerful yet intuitive **GUI**, BrainInsights democratizes advanced analytics, making these methods accessible to a broader scientific audience.

Furthermore, we directly addressed the critical challenges of reproducibility and scalability. By packaging the entire software stack into a single Singularity container, we ensure that an analysis is platform-independent and can be seamlessly scaled from a local workstation to a high-performance computing (HPC) cluster with guaranteed consistency. This containerized approach, combined with the framework’s inherent flexibility to handle complex multi-centric, multi-parametric, and longitudinal study designs, represents a significant step forward in providing a robust, adaptable, and user-friendly solution for the neuroimaging community.

Furthermore, its design is easily adaptable beyond MRI to other feature-extracted data types like EEG or PET.

### 4.5 Limitations

Despite its strengths, BrainInsights has several limitations that define its current scope and offer clear directions for future development. First, the framework operates on pre-processed, feature-extracted data and is not designed to perform initial raw image processing. A fundamental aspect of our scope is this reliance on derived features: users must first process raw image data using modality-specific and well established analysis-software before utilizing BrainInsights for statistical or machine learning analysis. Second, while scalable, certain components like MICE imputation and extensive Monte Carlo cross-validation can be computationally intensive, requiring significant time or HPC resources.

From a methodological standpoint, many clinical neuroimaging studies are constrained by small cohort sizes, which can increase the risk of model overfitting and reduce the generalizability of the findings. While our framework employs robust nested cross-validation and feature stability thresholds to mitigate these effects, the resulting bio-signatures still require external validation on large, independent datasets before they can be considered for clinical use.

From an analytical standpoint, a significant portion of the implemented machine learning algorithms are optimized for two-class (binary) classification problems, and the statistical modules do not yet include more complex methods like linear mixed-effects models for longitudinal analysis. This was a deliberate design choice based on most frequent clinical research requirements: the need for diagnostic differentiation and the prediction of binary treatment outcomes, such as responder vs. non-responders. By focusing on two-class problems, we ensure the generation of stable and interpretable bio-signatures that are directly applicable to clinical decision making. While this focus serves most current use cases, at the moment it does not fully support multi-class settings, regression for continuous clinical scores, or complex linear mixed-effects models for longitudinal analysis. However, many algorithms, like dimensionality reductions (cf. Figure 5) already fully support multi-class analyses. Finally, as a practical consideration, the entire ecosystem is currently built in **R**, which may be a limitation for researchers working primarily in other programing environments like Python. These limitations, however, provide a clear and exciting roadmap for the continued evolution of the BrainInsights framework.

### 4.6 Future directions

The current BrainInsights framework provides a robust foundation for an exciting and ambitious roadmap. Our future work will focus on three key areas: expanding the core analytical engine, broadening the scope of data integration, and driving toward clinical translation.

First, we will focus on expanding the core analytical toolkit. This involves incorporating a more extensive library of machine learning algorithms and moving toward Automated ML (AutoML), where the pipeline can intelligently search for the optimal modeling approach. Furthermore, we plan to extend the framework’s predictive scope to include multi-class classification and regression models, facilitating the analysis of continuous clinical scores and multi-stage disease progression. We will also add more advanced statistical methods, moving beyond group comparisons to include mixed-effects models for sophisticated longitudinal analysis and normative modeling, which would allow for comparing an individual patient against a population distribution like a “growth chart for the brain.” This evolution will be guided by community feedback and a commitment to enhancing our automated error-checking capabilities.

Second, we aim to significantly broaden the scope of data integration. A major long-term goal is to connect BrainInsights with raw imaging data pipelines (e.g., BIDS-compliant outputs), creating a seamless workflow from scanner to statistical insight. Beyond this, we plan to develop dedicated modules for multi-omics integration, particularly for imaging genetics or microbiome interactions. This would empower researchers to investigate how genetic or microbiome markers like polygenic risk scores manifest in the brain’s structure and function, linking the genome to the phenome within a single analytical environment. Furthermore, to optimize the handling of modalities with disparate noise profiles or information densities, we are investigating the transition from our current early fusion strategy toward late fusion or hybrid architectures. This would allow for independent feature processing before integrating individual model outputs into a final consensus bio-signature.

Perhaps the most significant future direction, however, is driving toward clinical translation. The key to this is building trust in model predictions. Having integrated XAI methods like SHAP to ML DaViz, our future work will focus on applying these insights. The key is to move beyond just identifying important features to explaining complex feature interactions. We also plan to develop visualization modules that aids specifically for clinical decision support. The goal is to explain why a model makes a prediction for a single patient—for instance, giving the clinician a score for patient’s likely treatment response. Moving from group-level research to these single-subject, clinically useful predictions is the central focus of our continued work.

## 5 Conclusion

In this paper, we introduced BrainInsights and validated it using previously published data. BrainInsights is an open-source, integrated software ecosystem designed to make advanced neuroimaging analysis more accessible, reproducible, and robust. By uniting traditional statistical testing, automated machine learning, and interactive visualization in a single framework, it empowers researchers to move fluidly between hypothesis-driven inquiry and data-driven discovery. The iterative workflow facilitated by BrainInsights is critical for validating findings, troubleshooting artifacts, and building the necessary confidence to identify robust bio-signatures from complex data. Ultimately, this framework represents a significant step forward in the effort to translate multi-modal data into meaningful and clinically relevant scientific insights.

## Data Availability

The data analyzed in this study is subject to the following licenses/restrictions: Patient. Requests to access these datasets should be directed to andreas.hess@fau.de.
