# Supplementary material for: BrainInsights: a comprehensive framework for pre-processing, analysis, and interpretation of neuroimaging data using traditional statistics and machine learning
Source: Front Neuroinform. 2026 Apr 15;20:1760583. doi: 10.3389/fninf.2026.1760583 (PMC13126547; doi:10.3389/fninf.2026.1760583)
Supplement: Supplementary file 1 [file Data_Sheet_1.docx]

# Supplement Methods:

## GUI Navigation Workflow:

The user journey through the GUI components typically follows this sequence:

1. Module Activation: Using the sidebar, the user selects a specific analytical task such as the options from MARIA like Basic Visualizations, Correlation plots, among others as showed in Figure S1A or options from ML-DaViz as shown in S1B.
2. Data Selection and Filtering: In the general options panel, For MARIA, Users then select the cohort (e.g., PreCePRA), specific centers and time points for analysis as shown in Figure S1C while in ML DaViz they select for which analysis from ML pipeline, i.e., based on which categorization, which feature selection method to be presented and which classifiers among others in this panel.
3. Parameter Configuration: The interface then updates with context specific controls, e.g., options regarding generating spider plots with ML DaViz as shown in Figure S1E or options regarding adjusting hyperparameters for t-SNE plots as shown in Figure S1F, such as perplexity and learning rates via intuitive checkboxes and sliders.
4. Interactive Execution and Export: Figure S1G, provides a look on options available to customize the plot. Options exist to edit the colors, title, X and Y labels depending on the plot as well as size of the text as well as the size and shape of the objects in the plot. Upon clicking "Update" or "Generate," the central window renders the analysis plot. Results are interactive, allowing for hover-over data inspection, and can be exported as high-resolution images (PNG/PDF) or structured tables (CSV/Excel) for publication.

## Detailed Data Pre-Processing Pipeline:

### Quality Assessment and Curation:

Before analysis, the framework performs a data curation step to handle potential errors, 'NaN' values, or non-numeric entries. This ensures the integrity and usability of the dataset.

### Missing Data Management:

Handling missing values effectively is a crucial pre-processing step. First, to prevent analytical bias, features with a high percentage of missing values can be optionally excluded. The user can define this threshold, though a rate above 30% is generally recommended for exclusion. Second, for the remaining features, the framework offers different imputation methodologies. The choice of imputation method is guided by recommendations based on the rate of missingness, as detailed in Table S2.

#### Imputation Methodologies:

##### **Replacing with zeros:** This method is fast but generally not recommended, as it can introduce inaccurate and misleading results.

- **Mean / Median Imputation:** These methods replace missing values with the mean or median of the respective feature. They are effective for numerical data but do not account for correlations between features.
- **Imputation using k-NN (k-Nearest Neighbors):** The k-nearest neighbors algorithm predicts missing values based on the nearest data points in the training set. Although computationally demanding, it offers higher accuracy than simpler methods but is sensitive to outliers.
- **Imputation using Multivariate Imputation by Chained Equations (MICE):** MICE is a robust and comprehensive method that fills in missing data multiple times and combines the results using statistical procedures like pooling estimates and Rubin's rules. It handles variables of different types (e.g. continuous, binary) and complexities, providing a robust approach to measuring the uncertainty of missing data.

### Data Scaling and Normalization:

Following imputation, the pipeline provides a suite of optional scaling and normalization methods to prepare the data for analysis. The available methods, their mathematical formulas, and typical applications are summarized in Table S3.

In addition to these standard methods, a specialized **Within-Subject Normalization** is available for longitudinal datasets. This approach applies a chosen scaling method to the repeated measurements within each participant., which is particularly useful for standardizing individual baseline differences in studies of disease progression or treatment effects. If a participant has only a single measurement, normalization is performed using the group average instead.

## Detailed Methodological Explanations:

**Cross Validation methods:**

The ML Pipeline is designed, with a flexible, nested validation strategy. The pipeline includes an option to initially split the data into a training/testing set and a final, held-out validation set (e.g. 90%/10%). This option is ideal for larger datasets where a final, untouched validation set is desired. However, this initial split is optional and was not used in the case studies presented due to limited sample sizes.

All model training and tuning are performed using one of the two robust, stratified cross-validation strategies to ensure unbiased model evaluation and preserve class balance.

- **Stratified K-fold Cross-Validation:** The combined training and test set is divided into k equal-sized folds (default k=5), with each fold maintaining the class propositions of the complete set. The model is trained on k-1 folds and tested on the remaining fold. This process is repeated k times, with each fold serving as the test set once, ensuring that all data points are used for both training and testing.
- **Stratified Monte Carlo Cross-Validation:** This method randomly splits the combined training and test set into training (default 75%) and test (default 25%) sets, maintaining the class balance in both subsets. This process is repeated multiple times (default 100) with different random seeds. This approach is particularly useful when the dataset is small or when there's a need to generate multiple random train-test splits.

**Feature Selection algorithms:**

- ***Boruta****:* The Boruta algorithm^22^ compares feature importance in the original dataset with the significance of shadow features created by random permutations using a random forest classifier. The shadow features have no meaningful relationship with the target variable, serving as a baseline for comparison. Features that are more important than their corresponding shadow features are considered relevant and are retained, while features with lower importance are deemed unimportant and removed. Boruta provides, compared to SPLSDA, a more robust and unbiased automatic feature selection process.
- ***Sparse partial least square discriminant analysis (sPLS-DA):*** The Sparse partial least square discriminant analysis (sPLS-DA)^23^ combines the principles of Partial Least Squares (PLS) regression^44^ and LASSO (Least Absolute Shrinkage and Selection Operator) methods^45^. First, sPLS-DA performs a PLS decomposition to find latent variables that explain the maximum covariance between the predictor variables (features) and the class labels (response variable). Then, it introduces a sparsity constraint to the loading weights of the PLS components, inciting many loading weights to be precisely zero. This sparsity constraint leads to selecting a subset of features with the most discriminative power for later classification. The main advantage of sPLS-DA lies in its ability to identify relevant features while handling the curse of dimensionality, which occurs when the number of features exceeds the number of samples. By selecting a sparse subset of features, sPLS-DA reduces the risk of overfitting and improves the generalization performance of classification models.
- ***Recursive Feature elimination:*** Recursive Feature Elimination (RFE)^24^ is a feature selection technique that recursively fits a model and ranks the features based on their impact on its performance. The least important features are eliminated in each iteration until a desired subset of relevant features is obtained. RFE helps improve model generalization, reduce overfitting, and enhance predictive accuracy by focusing on the most informative features. It is beneficial for high-dimensional data, where the number of features is much larger than the number of samples.

## Glossary of Terms:

AN: Anorexia Nervosa

ANOVA: Analysis of Variance

ANCOVA: Analysis of Covariance

AUC: Area Under the Curve

BA: Balanced Accuracy

BH: Benjamini-Hochberg (p-value correction method)

BMI: Body Mass Index

BOLD: Blood-Oxygen-Level Dependent

BY: Benjamini-Yekutieli (p-value correction method)

CNS: Central Nervous System

CSV: Comma Separated Values

DBM: Deformation-based morphometry pipeline

DTI: Diffusion Tensor Imaging

EEG: Electroencephalography

FDR: False Discovery Rate (p-value correction method)

fMRI: functional Magnetic Resonance Imaging

GLM: Generalized Linear Model

GUI: Graphical User Interface

HC: Healthy Controls

HPC: High-Performance Computing

IQR: Interquartile Range

k-NN: k-Nearest Neighbors

LASSO: Least Absolute Shrinkage and Selection Operator

LDA: Linear Discriminant Analysis

MANOVA: Multivariate Analysis of Variance

MAR: Missing at Random

MARIA: MAgnetic Resonance Imaging data Analysis and inspection tool

MCAR: Missing Completely At Random

MICE: Multivariate Imputation by Chained Equations

ML: Machine Learning

ML DaViz: Machine Learning Analysis and Data Visualization tool

ML Pipeline: Machine Learning Pipeline

MRI: Magnetic Resonance Imaging

NaN: Not a Number

PCA: Principal Component Analysis

PET: Positron Emission Tomography

PLS: Partial Least Squares

RDS: R Data Structure

RFE: Recursive Feature Elimination

ROC: Receiver Operating Characteristic

ROI: Regions of Interest

SEM: Standard Error of Measurement

SIMCA: Soft Independent Modelling of Class Analogy

sPLS-DA: Sparse Partial Least Squares Discriminant Analysis

SVM: Support Vector Machine

SYLK: Symbolic Link (file format)

t-SNE: t-Distributed Stochastic Neighbor Embedding

UMAP: Uniform Manifold Approximation and Projection

VBM: Voxel-Based Morphometry

XGBoost: Extreme Gradient Boosting

## Tables:

| Framework | Input | Output |
| --- | --- | --- |
| Data Import | - Feature-extracted neuroimaging data in tabular formats (.xlsx, .csv or .sylk) formats derived from external pipelines (e.g. Freesurfer, VBM) | - Standardized **subject-by-feature matrices** saved in lightweight .rds or high speed feather formats. |
| MARIA | - Standardised N x P subject by feature matrix (.rds/.feather) - **Group assignment file**: An excel file used to dynamically define or update sub-cohorts without re-preprocessing the entire dataset. | - Publication-quality plots (.pdf/.png) - Statistical summary tables (.csv/.xlsx) |
| ML Pipe | - Standardised N x P subject by feature matrix (.rds/.feather) - Group assignment file - Configuration file: A user-defined YAML file specifying data splitting strategies, cross-validation methods, feature selection algorithms, and model hyperparameters. | - Comprehensive RDS files containing: random seeds for reproduction, model performance metrics (e.g. accuracy, AUC, F1-scores), list of selected features and class prediction and probabilities for each participant (For ROC generation). |
| ML DaViz | - Standardised N x P subject by feature matrix (.rds/.feather) - .rds output file generated by ML Pipe | - Publication-quality plots (.pdf/.png) - Selected features summary tables (.csv/.xlsx) |

Table S1: Input/Output Specifications for the BrainInsights Modular Components. This table details the data requirements and file formats necessary for the independent operation of the Data Import, MARIA, ML Pipeline, and ML DaViz modules.

| **Missing Rate** | **Recommendation** | **Rationale** |
| --- | --- | --- |
| 0% | No action needed | Complete data |
| <5% | Simple imputation | Missing Completely at Random (MCAR) likely, minimal impact |
| 5-15% | Advanced imputation | Consider Missing at Random (MAR) mechanisms |
| 15-30% | Evaluate importance | Potential bias introduction |
| >30% | Consider dropping | High risk of bias |

Table S2 Recommendations for Handling Missing Data: The table outlines the pipeline’s recommended strategies for managing missing data based on the percentage of missing values in a feature. These guidelines are designed to minimize bias while retaining valuable data.

| **Scaling Method** | **Formula** | **Application** | **Recommendation** | **Rationale**  **/Advantage** |
| --- | --- | --- | --- | --- |
| Standard Scaling | (x−μ)/σ | Normally distributed features | VBM and DTI measures | Preserves distributional properties while standardizing variance |
| Robust Scaling | (x−median)/IQR | Datasets with outlier presence | FreeSurfer morphometric measures | Reduced sensitivity to extreme values |
| MinMax Scaling | (x−min)/(max−min) | Bounded measurement ranges | Functional connectivity matrices | Values constrained to [0,1] interval |
| Logarithmic Transformation | log(x) or log(x+1) | Addresses positive skew distributions | Suitable for ratio-scale measurements | Transforms skewed data to be more symmetrical. |
| Box-Cox Transformation | Optimal power transform | Non-normal distributions | Comprehensive normality approximation | Automated optimal λ parameter determination. Applicable to various distributional shapes. |

Table S3: Data Scaling and Normalization Methods. Summary of primary scaling and normalization methods available in pre-processing pipeline. The table includes the mathematical formula for each method, its typical application, and the rationale for its use. IQR refers to the interquartile range.

| Case Study | Dataset Name | Population and Sample Size (N) | Modalities | Primary objective |
| --- | --- | --- | --- | --- |
| 1: Standard Workflow | Neurotrition | N = 61 (13 Adult AN, 15 Adult HC, 17 Young AN, 16 Young HC)  AN – Anorexia  HC – Healthy Controls | T1 MRI, rs-fMRI, Clinical | Diagnostic classification of Anorexia Nervosa (AN) from healthy controls (HC) |
| 2: Hypotheses Testing | PreCePRA | N = 139 Patients with Rheumatoid Arthritis (randomly assigned to high/low CNS activation or placebo) | BOLD and rs-fMRI, T1, T2, DTI, Clinical | Predicting clinical response to anti-TNF treatment using functional-pain related markers |
| 3: Iterative Validation | Crohn’s disease study | 11 CD patients and 11 healthy controls | BOLD and rs-fMRI, T1, T2, Clinical | Cross-method verification and biological validation of findings |

Table S4: Summary of datasets from case studies and study objectives of framework validation.

## Figure Legends and Captions:

*Figure S1: Representative examples of functional modules within the BrainInsights GUI ecosystem ( MARIA and ML DaViz). (A-B) Modular sidebars for selecting specific analytical tabs. (C ) Data scoping interface for filtering cohorts, centers and measurements illustrated with the PreCePRA study parameters. (D, E, F) Menus for choosing plot types, options for definig visualization constraints for a spider plot and optimizing hyperparameters for a t-SNE plot. (G) Advanced configuration panels for publication-ready plot styling.*
