# Supplementary material for: BrainInsights: a comprehensive framework for pre-processing, analysis, and interpretation of neuroimaging data using traditional statistics and machine learning
Source: Front Neuroinform. 2026 Apr 15;20:1760583. doi: 10.3389/fninf.2026.1760583 (PMC13126547; doi:10.3389/fninf.2026.1760583)
Supplement: Supplementary file 2 [file Data_Sheet_2.pdf]

```
common_settings:
  run_on_cluster: no
  what_to_run:
  - boruta_ml
  - splsda_ml
  use_init_file: no
  cluster_wall_time: '24:00:00'
  overwrite_settings: yes
  overview_file: overview_V1.xlsx
  linux_working_directory: /home/hpc/mfpt/mfpt002h/Desktop/NDA/
MLPipe
  main_dataset_path: /../data/PreCePRA/
PreCePRA_Clinical_Without_diff_DAS_scores.rds
  group_assignment_file_path: /../data/PreCePRA/group_info_V10.xlsx
  function_folder_path: functions
  iterations: 100.0
  iterations_per_job: 100.0
splsda_tuning_settings:
  tune: yes
  ncomp: 6.0
  list_keep_x:
  - 1.0
  - 2.0
  - 3.0
  - 4.0
  - 5.0
  - 10.0
  - 20.0
  - 50.0
  - 100.0
  - 500.0
  - 1000.0
  folds: 4.0
  nrepeat: 100.0
  validation: Mfold
  dist: max.dist
  measure: BER
  cpus: 4.0
  default_n_comp: 1.0
  default_keep_x: 5.0
splsda_feature_selection_settings:
  ncomp: 2.0
  keep_x:
  - 10.0
  - 10.0
  max_iterations: 1000.0
  scale: yes
  near_zero_var: yes
boruta_feature_selection_settings:
  p_value: 0.05
  max_runs: 500.0
  do_trace: 0.0
  get_importance: getImpRfZ
ml_settings:
```

```
  common_settings:
    initial_seed: 42.0
    iterations: 100.0
    training_sample_size: 80.0
    classification_algorithms:
    - RF
    - catboost
    - neuralnet
    - XGBoost
    - SVM
    - LDA
    - rFerns
    - RSimca
    - GLM
    - splsda
    useUMAP: no
    leave_out_features:
    - centre
    - subject_name
    - measurement
    - name
    - participant_name
explicit_hyperparameters:
  Random_Forest:
    ntree: 1000.0
    importance: yes
  SVM_Radial:
    sigma: 0.05
    C: 2.2
    metric: Accuracy
  XGBoost:
    objective: binary:hinge
    eta: 0.03
    max_depth: 6.0
    eval_metric: auc
    nrounds: 100.0
  Neural_Network:
    size: 10.0
    decay: 0.1
    MaxNWts: 200000.0
  rFerns:
    depth: 5.0
    ferns: 1000.0
    importance: none
  sPLS_DA_Classification:
    ncomp: 1.0
    max_iter: 1000.0
    scale: yes
    near_zero_var: yes
  Catboost:
    iterations: 500.0
    learning_rate: 0.03
    depth: 6.0
    trControl_method: boot
```

```
LDA:
  method: moment
  trControl_folds: 10.0
Naive_Bayes:
  laplace: 0.0
  usekernel: no
  adjust: 1.0
GLM:
  family: binomial
  trControl_folds: 10.0
RSimca:
  method: RSimca
  trControl_folds: 10.0
```
