# Supplementary figures and images for: BrainInsights: a comprehensive framework for pre-processing, analysis, and interpretation of neuroimaging data using traditional statistics and machine learning
Source: Front Neuroinform. 2026 Apr 15;20:1760583. doi: 10.3389/fninf.2026.1760583 (PMC13126547; doi:10.3389/fninf.2026.1760583)

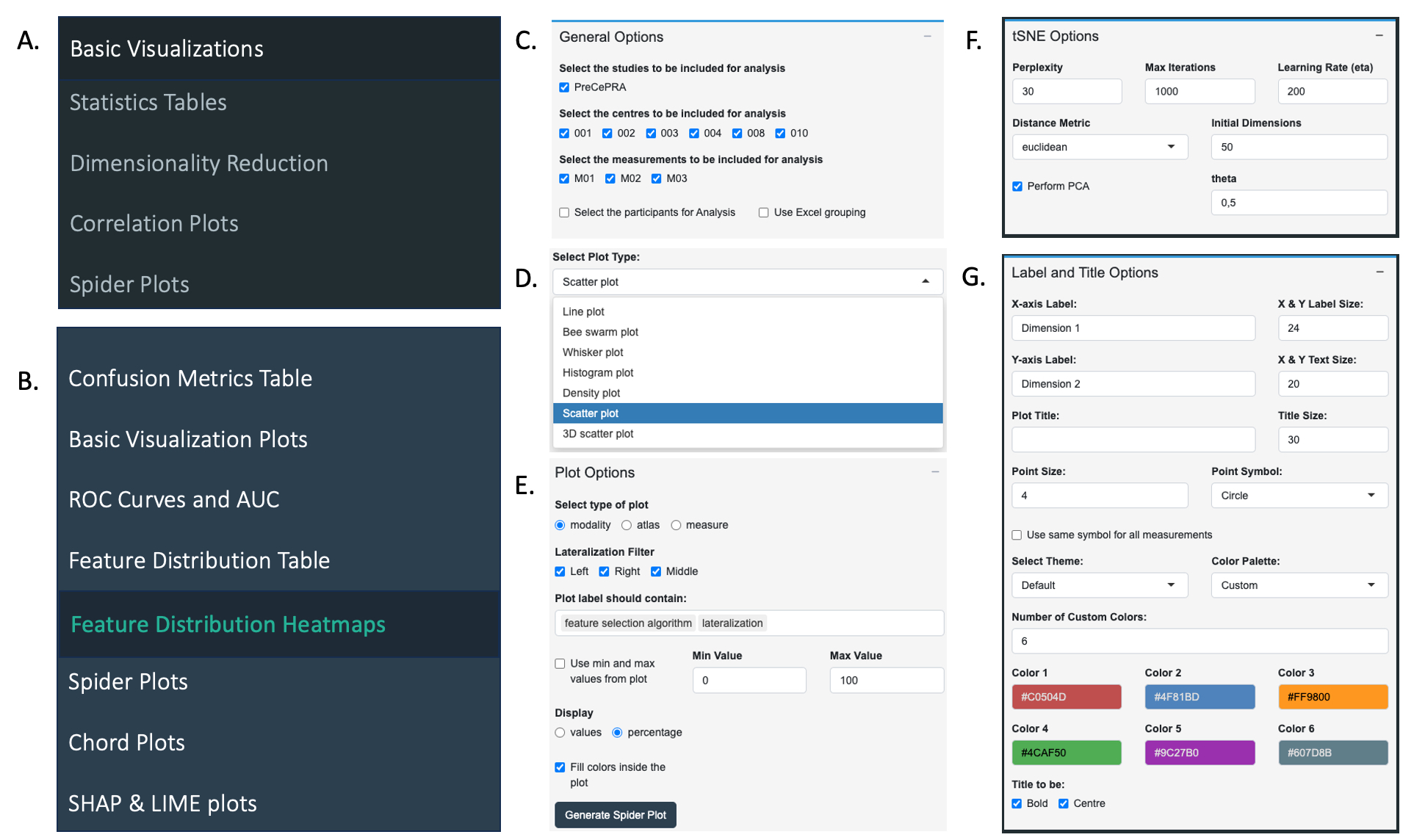

Supplement: Supplementary file 3 [file Image_1.jpeg]
